# Tenacibactins K–M, cytotoxic siderophores from a coral-associated gliding bacterium of the genus *Tenacibaculum*

**DOI:** 10.3762/bjoc.18.12

**Published:** 2022-01-13

**Authors:** Yasuhiro Igarashi, Yiwei Ge, Tao Zhou, Amit Raj Sharma, Enjuro Harunari, Naoya Oku, Agus Trianto

**Affiliations:** 1 Biotechnology Research Center, Toyama Prefectural University, Imizu, Toyama 939-0398, Japan; 2 Faculty of Fisheries and Marine Sciences, Diponegoro University, Tembalang Campus, St. Prof. Soedarto SH., Semarang 50275, Central Java, Indonesia

**Keywords:** desferrioxamine, marine obligate bacterium, MS/MS analysis, tenacibactin, *Tenacibaculum*

## Abstract

HPLC/DAD-based chemical investigation of a coral-associated gliding bacterium of the genus *Tenacibaculum* yielded three desferrioxamine-class siderophores, designated tenacibactins K (**1**), L (**2**), and M (**3**). Their chemical structures, comprising repeated cadaverine–succinic acid motifs terminated by a hydroxamic acid functionality, were elucidated by NMR and negative MS/MS experiments. Compounds **1**–**3** were inactive against bacteria and a yeast but displayed cytotoxicity against 3Y1 rat embryonic fibroblasts and P388 murine leukemia cells at GI_50_ in submicromolar to micromolar ranges. Their iron-chelating activity was comparable to deferoxamine mesylate.

## Introduction

Marine organisms continue to be a prolific resource of new bioactive natural products that are applicable to pharmaceutical purposes. Especially, marine invertebrate-associated microbes are emerging as one of the hotspots for these molecules [1]. Marine invertebrates, including corals, have a sessile habit and thus are vulnerable to environmental stresses including predation and competition. They instead harbour diverse and abundant microbes on their body surface or in the tissues [2–3] and are believed to utilize secondary metabolites from the symbionts as protectants from attacks by predators, competitors, or pathogens. The ecological functions as such make marine microorganisms an attractive resource of new therapeutics, which are not found from terrestrial bioresources [4–6].

While a large majority of marine microbe-derived natural products are from fungi and actinomycetes, less attention has been paid to non-actinomycetal bacteria [6–9]. Particularly, secondary metabolites from Gram-negative bacteria are still quite limited, despite the predominance of this group in the marine environment [10–11].

**Figure 1:**
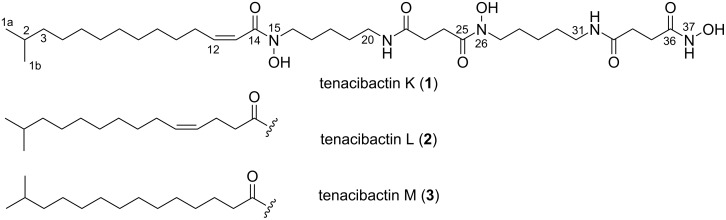
Structures of tenacibactins K–M (**1**–**3**).

The genus *Tenacibaculum* belongs to the family *Flavobacteriaceae* within the phylum *Bacteroidetes*. Members of this genus are Gram-negative, aerobic, motile by gliding, and commonly isolated from marine environments [12–15]. Several *Tenacibaculum* species are identified as fish pathogens, among which *T. maritimum* has been the most well-studied as an etiological agent of tenacibaculosis, a skin ulcer disease for marine fish [16]. At present, only two reports are available on the secondary metabolites from this genus [17–18]. In our continuing search for bioactive compounds from underexplored marine bacteria [19–21], a *Tenacibaculum* strain, isolated from a stony coral, was found to produce three metabolites, which turned out to be new cytotoxic hydroxamate-class siderophores, tenacibactins K–M (**1**–**3**, Figure 1).

## Results and Discussion

The producing strain C16-1 was isolated from a scleractinian coral of the genus *Favia* and was identified as a member of the genus *Tenacibaculum* on the basis of 16S rRNA gene sequence similarity. The same strain was cultured in three different seawater-based media, and butanolic extracts of the fermented cultures were subjected to HPLC/DAD analysis, which detected several unknown metabolites not present in our in-house UV database, showing UV end-absorption in the culture extract of A11M seawater medium. Purification of these peaks resulted in the isolation of tenacibactins K (**1**), L (**2**), and M (**3**).

Compound **1** was obtained as a pale brown powder. HR–ESITOFMS analysis confirmed the molecular formula of **1** to be C_33_H_61_N_5_O_8_ based on a deprotonated molecular ion [M − H]^−^ at *m*/*z* 654.4449 (Δ + 0.2 mmu for C_33_H_60_N_5_O_8_) and a sodium adduct [M + Na]^+^ at *m/z* 678.4412 (Δ + 0.0 mmu for C_33_H_61_N_5_O_8_Na). Analysis of ^13^C NMR and HSQC spectroscopic data obtained in DMSO-*d*_6_ established the presence of five carbonyl carbons (δ_C_ 166.2, 168.6, 171.0, 171.5, 172.0), two sp^2^ methines (δ_C_ 119.8, 144.5), one sp^3^ methine (δ_C_ 27.5), two magnetically equivalent doublet methyls (δ_C_ 22.6/δ_H_ 0.83 for six protons) (Table 1), along with many overlapping deshielded and shielded methylenes.

**Table 1:** ^1^H and ^13^C NMR data for tenacibactin K (**1**) in DMSO-*d*_6_.

position	δ_C_^a^, type	δ_H_, mult (*J* in Hz)^b^	HMBC^b,c^

1a	22.6, CH_3_	0.83, d (6.6)	1b, 2, 3
1b	22.6, CH_3_	0.83, d (6.6)	1a, 2, 3
2	27.5, CH	1.48^d^	1a, 1b, 3, 4
3	38.56, CH_2_	1.12, m	1a, 1b, 2, 4, 5
4	26.9, CH_2_	1.22^d^	
5	29.4, CH_2_	1.22^d^	
6	28.88^e^, CH_2_	1.20 to ≈1.25^d^	
7	28.95^e^, CH_2_	1.20 to ≈1.25^d^	
8	29.1^e^, CH_2_	1.20 to ≈1.25^d^	
9	29.2^e^, CH_2_	1.20 to ≈1.25^d^	
10	28.7, CH_2_	1.33, m	12^f^
11	28.3, CH_2_	2.49, m	12^f^, 13^f^
12	144.5, CH	5.98, dt (11.4, 7.4)^g^	14^f^
13	119.8, CH	6.30, d (11.4)	11^f^, 14^f^
14	166.2, C		
16	46.7, CH_2_	3.48^d^	14, 17, 18
17	26.1, CH_2_	1.52^d^	16, 18, 19
18	23.6, CH_2_	1.21^d^	
19	28.8, CH_2_	1.37^d^	17, 18, 20
20	38.52, CH_2_	3.00^d^	18, 19, 22
21-NH		7.79, t (4.9)	20, 22
22	171.5, C		
23	30.1, CH_2_	2.26, t (7.4)	22, 24, 25
24	27.7, CH_2_	2.57, t (7.4)	22, 23, 25
25	172.0, C		
27	47.2, CH_2_	3.45^d^	25, 28, 29
28	26.1, CH_2_	1.50^d^	27, 29, 30
29	23.6, CH_2_	1.21^d^	
30	28.8, CH_2_	1.37^d^	28, 29, 31
31	38.52, CH_2_	2.99^d^	29, 30, 33
32-NH		7.81, t (5.2)	31, 33
33	171.0, C		
34	30.7, CH_2_	2.28, t (7.0)	33, 35, 36
35	28.0, CH_2_	2.16, t (7.1)	33, 34, 36
36	168.6, C		
NH or OH		8.71, brs	
NH or OH		9.71, brs	
NH or OH		10.39, brs	

^a^Referenced to a septet peak of DMSO-*d*_6_ at 39.5 ppm. ^b^Referenced to a quintet peak from residual DMSO-*d*_6_ at 2.50 ppm. ^c^From proton to indicated carbons. ^d^Overlapped. ^e^Interchangeable. ^f^Observed in a mixed solvent CDCl_3_/CD_3_OD 3:7. ^g^Coupling constants acquired at 50 °C.

**Figure 2:**
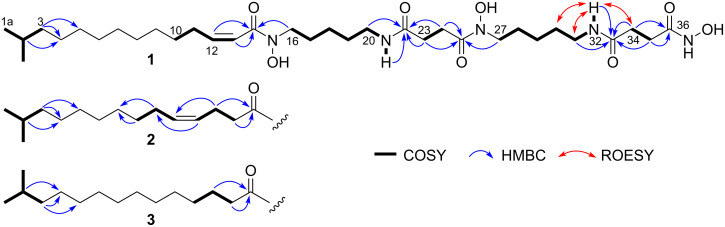
Key 2D-NMR correlations for **1**–**3**.

A ^1^H NMR spectrum showed two olefinic resonances (δ_H_ 5.98 and 6.30) with the lowest signal intensities and others in two-to-six-fold higher intensities than these, indicating the presence of duplicated substructures. Indeed, a careful analysis of a COSY spectrum identified a pair of five-methylene fragments (H16–H20 and H27–H31) with deshielded protons/carbons at both ends (C16: δ_H_ 3.48/δ_C_ 46.7; C20: δ_H_ 3.00/δ_C_ 38.52; C27: δ_H_ 3.45/δ_C_ 47.2; C31: δ_H_ 2.99/δ_C_ 38.52), and further coupling of these fragments to exchangeable protons at δ_H_ 7.79 or 7.81, leading to the assignment of two cadaverine moieties (Figure 2). Similarly, another pair of two-methylene fragments (H23–H24 and H34–H35) were found, which respectively displayed HMBC correlations to two carbonyl carbons (δ_C_ 171.5 and 172.0; 168.6 and 171.0), thus establishing two succinic acid moieties (C22–C25; C33–C36, Figure 2). Connection of these substructures via the amide bonds with an alternate alignment of cadaverine and succinic acid was verified by HMBC correlations from the amide protons to the adjacent carbonyl carbons (H21/C22 and H32/C33) and the aminomethylene proton to another carbonyl carbon (H27/C25).

**Figure 3:**
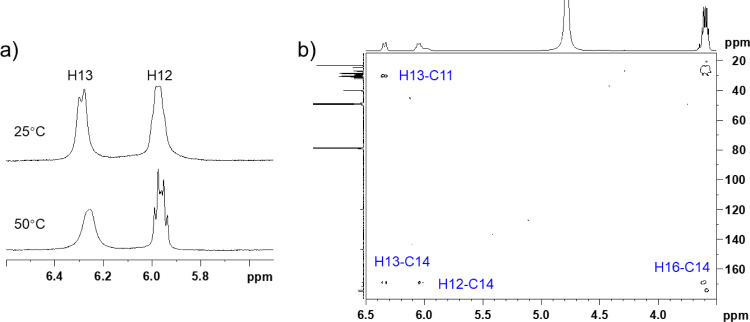
(a) Partial ^1^H NMR spectra of **1** at 25 and 50 °C in DMSO-*d*_6_; (b) magnified HMBC spectrum of **1** at 25 °C in CDCl_3_/CD_3_OD 3:7.

The remaining COSY correlations assembled a 1,2-disubstituted double bond with a two-methylene extension (C10–C13) and an isobutyl fragment (C1a,b–C3) from the rest of the molecular parts. Quite uniquely, both of the olefinic proton resonances (H12 and H13) were broadened at 25 °C (Figure 3a). However, upon heating to 50 °C, H12 split into doublet-triplet, which allowed the extraction of ^3^*J*_H12,H13_ = 11.4 Hz to deduce a *cis* configuration. H13, in contrast, broadened more severely at the raised temperature, which was eventually attributed to the accelerated dissociation of the neighbouring hydroxamate group in a polar aprotic solvent, DMSO-*d*_6_. The isobutyl fragment showed HMBC correlations to two methylenic carbons δ_C_ 26.9 (C4) and 29.4 (C5), which provided an isohexyl fragment (Table 1). The remaining four methylenes (H6, H7, H8, H9) were not assignable from the NMR data due to signal overlapping, but were expected to be placed between the isohexyl and the alkenyl fragments, thus establishing an isopentadecenoyl moiety. The connectivity between this aliphatic chain to the tandem succinylcadaverine unit was not proven due to the lack of relevant HMBC correlations in DMSO-*d*_6_. However, when measured in a mixed solvent (CDCl_3_/CD_3_OD 3:7), the peak shape of H13 was sharpened and HMBC correlations from both of the olefinic protons and the aminomethylene H16 to a carbonyl carbon (C14: δ_C_ 166.2) were detected, which joined the C_15_-acyl unit to the cadaverine end (Figure 3b).

The structure so far assembled left H_4_NO_3_ yet to be assigned. A structural similarity of **1** to the known microbial siderophores containing the cadaverine-succinate motifs was suggestive of the presence of *N*-hydroxy groups in **1**. Among the five amide bonds, amide protons were present at N21 and N32, thereby leaving N15, N26, and N37 as the hydroxylation sites. This assignment was supported by the ^13^C NMR chemical shifts. Within each cadaverine moiety, the ^13^C chemical shifts for the methylenes adjacent to the *N*-hydroxyamide group (C16: δ_C_ 46.7; C27: δ_C_ 47.2) were obviously larger than the methylenes adjacent to the amide group (C20: δ_C_ 38.52; C31: δ_C_ 38.52), consistent with the reported data for avaroferrin [22], bisucaberins [18], and nocardamines [23]. However, this trend is inversed in the hydroxamic acid terminus. The methylene carbon C35 adjacent to the hydroxamic acid group showed a smaller chemical shift (δ_C_ 28.0). The positional assignment of C34 and C35 was made by a ROESY correlation observed between H34 and 32-NH (Figure 2).

**Figure 4:**
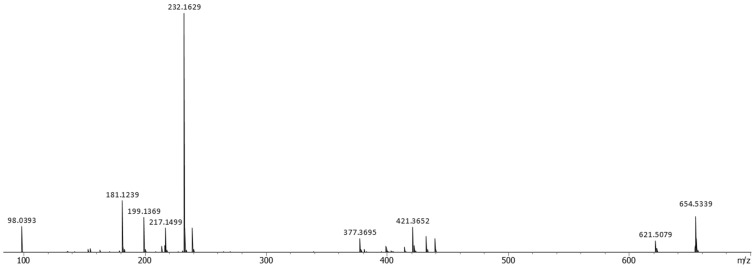
MS/MS spectrum of **1** acquired on a quadrupole time-of-flight mass spectrometer in the negative ion mode.

**Scheme 1:**
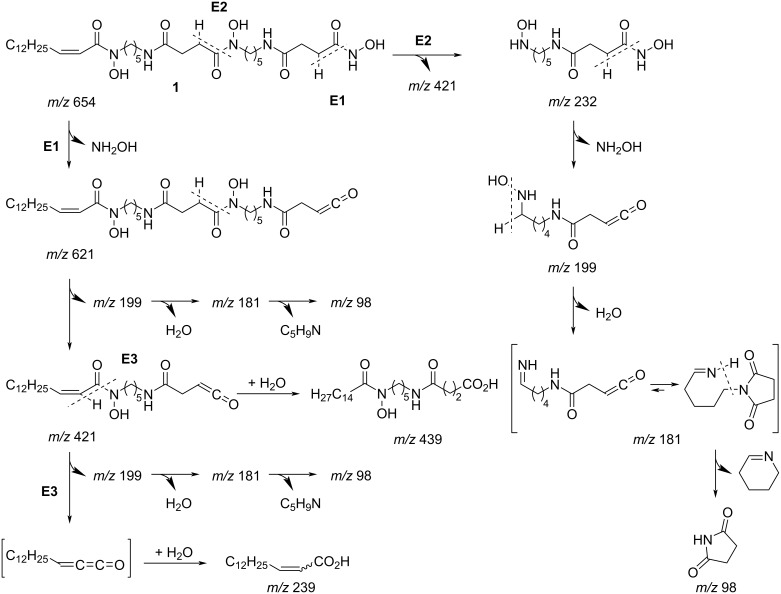
MS/MS fragmentation pathway for compound **1**.

To verify the structure deduced from the NMR analysis, an MS/MS analysis was conducted [24] (Figure 4). In the negative ion mode, a precursor ion *m*/*z* 654 underwent sequential eliminations at every hydroxamate C–N bond, giving rise to ketene-terminated product ions at *m*/*z* 621 and 421, which supported the position of hydroxylation at N37 and N26 and chain lengths of each cadaverine/succinic acid module (Scheme 1, paths E1 and E2). The third elimination product, C_15_-ketene (structure in square brackets, Scheme 1) was not observed, but a pentadecenoate anion, appearing at *m*/*z* 239, warranted the existence of fragmentation path E3 and also the chain length of the acyl unit. Hydration of ketene to give carboxylate was also detected as an ion at *m*/*z* 439*.* The fragment ions *m*/*z* 232, 199, 181, and 98 were commonly detected in the MS/MS spectra for compounds **1**–**3**, which appeared to be derived from the right half of the molecule by sequentially losing hydroxyamine, water, and tetrahydropyridine after formation of *N*-alkylated succinimide to end up as a succinimide anion. Based on these analyses, the structure of **1** was unambiguously established.

Compound **2** gave molecular ions at almost the same *m*/*z* as compound **1** in the HR–ESITOFMS analysis, revealing an identical molecular formula to **1**. While no significant difference was seen between the MS/MS spectra of compounds **1** and **2** (Figure 4, and Figure S19 and Scheme S20 in Supporting Information File 1), the ^1^H NMR spectrum of the latter exhibited coalesced olefinic signals in a deshielded region (δ_H_ 5.33) and two additional methylene resonances (H12 and H13) at δ_H_ 2.19 and 2.36, implying translocation of the double bond in the acyl portion (Table 2). The analysis of the COSY spectrum connected the above described methylenes into a bismethylene fragment, which in turn showed HMBC correlations to a carbonyl carbon (C14: δ_C_ 172.2) and two olefinic carbons (C10: δ_C_ 130.2; C11: δ_C_ 128.9), revealing the site of unsaturation at a γ,δ-position (Figure 2 and Table 2). The double bond geometry was determined to be *cis* on the basis of the chemical shifts of the allylic carbons (C12: δ_C_ 22.3, C9: δ_C_ 26.6) [25], which are closer to those of a (*Z*)-isomer (δ_C_ 22.5 and 27.2) [26] than those of an (*E*)-isomer (δ_C_ 28.8 and 34.8 ppm) [27]. Thus, the structure of compound **2** was determined to be a double-bond regioisomer of **1**.

**Table 2:** ^1^H and ^13^C NMR data for tenacibactins L (**2**) and M (**3**) in DMSO-*d*_6_.

	**2**				**3**		
		
position	δ_C_^a^, type	δ_H_, mult (*J* in Hz)^b^	HMBC^c^		δ_C_^a^, type	δ_H_, mult (*J* in Hz)^b^	HMBC^c^
		
1a	22.6, CH_3_	0.83, d (6.6)	1b, 2, 3		22.6, CH_3_	0.83, d (6.6)	1b, 2, 3
1b	22.6, CH_3_	0.83, d (6.6)	1a, 2, 3		22.6, CH_3_	0.83, d (6.6)	1a, 2, 3
2	27.5, CH	1.48^d^	1a, 1b, 3, 4		27.5, CH	1.48^d^	1a, 1b, 3, 4
3	38.52, CH_2_	1.12, m	1a, 1b, 2, 4, 5		38.54, CH_2_	1.12, m	1a, 1b, 2, 4, 5
4	26.8, CH_2_	1.22^d^			26.8, CH_2_	1.22^d^	
5	29.3, CH_2_	1.22^d^			29.4, CH_2_	1.22^d^	
6	29.0, CH_2_	1.24^d^			28.88^e^, CH_2_	1.20 to ≈1.25^d^	
7	28.7, CH_2_	1.23^d^			28.92^e^, CH_2_	1.20 to ≈1.25^d^	
8	29.1, CH_2_	1.29, m	7, 9		29.07^e^, CH_2_	1.20 to ≈1.25^d^	
9	26.6, CH_2_	1.98, dt (6.0, 6.7)	7, 8, 10, 11		29.09^e^, CH_2_	1.20 to ≈1.25^d^	
10	130.2, CH	5.33^d^	8, 9, 12		29.13^e^, CH_2_	1.20 to ≈1.25^d^	
11	128.9, CH	5.33^d^	9, 12, 13		29.0, CH	1.22^d^	
12	22.3, CH_2_	2.19, dt (6.4, 7.4)	10, 11, 13, 14		24.3, CH_2_	1.45^d^	11, 13, 14
13	32.0, CH_2_	2.36, t (7.7)	11, 12, 14		31.8, CH_2_	2.31, t (7.1)	11, 12, 14
14	172.2, C				172.8, C		
16	47.2, CH_2_	3.44^d^	14, 17, 18		47.0, CH_2_	3.44^d^	14, 17, 18
17	26.1, CH_2_	1.48^d^	16, 18, 19		26.1, CH_2_	1.48^d^	16, 18, 19
18	23.6, CH_2_	1.20^d^			23.6, CH_2_	1.20^d^	
19	28.8, CH_2_	1.37^d^	17, 18, 20		28.8, CH_2_	1.37^d^	17, 18, 20
20	38.51, CH_2_	2.99^d^	18, 19, 22		38.52, CH_2_	2.99^d^	18, 19, 22
21-NH		7.78, t (5.2)	20, 22			7.80^d^	22
22	171.5, C				171.5, C		
23	30.0, CH_2_	2.26, t (7.1)	22, 24, 25		30.0, CH_2_	2.26^d^	22, 24, 25
24	27.6, CH_2_	2.57, t (7.0)	22, 23, 25		27.7, CH_2_	2.57, t (7.1)	22, 23, 25
25	172.0, C				172.1, C		
27	47.2, CH_2_	3.44^d^	25, 28, 29		47.2, CH_2_	3.44^d^	25, 28, 29
28	26.1, CH_2_	1.48^d^	27, 29, 30		26.1, CH_2_	1.48^d^	27, 29, 30
29	23.6, CH_2_	1.20^d^			23.6, CH_2_	1.20^d^	
30	28.8, CH_2_	1.37^d^	28, 29, 31		28.8, CH_2_	1.37^d^	28, 29, 31
31	38.51, CH_2_	2.99^d^	29, 30, 33		38.52, CH_2_	2.99^d^	29, 30, 33
32-NH		7.81, t (4.9)	31, 33			7.80^d^	33
33	171.0, C				171.0, C		
34	30.7, CH_2_	2.28, t (7.0)	33, 35, 36		30.7, CH_2_	2.28^d^	33, 35, 36
35	28.0, CH_2_	2.16, t (7.1)	33, 34, 36		28.0, CH_2_	2.16, t (7.2)	33, 34, 36
36	168.6, C				168.6, C		
NH or OH		8.71, brs				8.73, brs	
NH or OH		9.69, brs				9.69, brs	
NH or OH		10.39, brs				10.38, brs	

^a^Referenced to a septet peak of DMSO-*d*_6_ at 39.5 ppm. ^b^Referenced to a quintet peak from residual DMSO-*d*_6_ at 2.50 ppm. ^c^From proton to indicated carbons. ^d^Overlapped. ^e^Interchangeable.

The molecular formula of **3**, determined to be C_33_H_63_N_5_O_8_ based on a deprotonated molecular ion at *m/z* 656.4604 (Δ 0.0 mmu for C_33_H_62_N_5_O_8_) and a sodium adduct ion at *m/z* 680.4567 (Δ **−** 0.2 mmu for C_33_H_63_N_5_O_8_Na), was larger by two hydrogen atoms than that of compound **1** or **2**. Indeed, olefinic resonances were absent in the NMR spectra and MS/MS fragment ions from the left half of the molecule were larger by 2 mass units than those for compounds **1** and **2** (*m/z* 623, 441, 423, and 241, Figure S28 and Scheme S29 in Supporting Information File 1), supporting a saturated fifteen-carbon acyl moiety in compound **3**. This assignment was corroborated by substantially the same NMR data for the remaining part of **1**–**3**. Thus, **3** was concluded to be a saturated congener of compounds **2** and **3**.

Tenacibactins K−M (**1**–**3**) are new members of desferrioxamine-type hydroxamate siderophores [28]. The preceding congeners are tenacibactins A–D produced by *Tenacibaculum* sp. [18] and tenacibactins E–J produced by *Streptomyces* sp. [29]. Siderophores of this class are produced by both Gram-positive and -negative bacteria and have a linear or macrocyclic backbone [23,30] composed of alternately arranged cadaverine or putrescine and succinic acid modules with *N*-hydroxylation at every other amide bond. Modifications of these core structures include internal hydroxylation [30], terminal blocking by acylation [29,31–32], formation of sugar ester [33], imine oxide [34], oxime [35], or functional group transformation into a hydroxy [33] or nitro group [35]. To the best of our knowledge, compounds **1**–**3** are the first to have a hydroxamic acid terminus. Similar to the related compounds such as nocardichelins [31] and MBJ-0003 [32], compounds **1**–**3** did not show appreciable antimicrobial activity against bacteria or a yeast (see Experimental) at 50 μg/mL but exhibited cytotoxicity against 3Y1 rat embryonic fibroblasts and P388 murine leukemia cells (Table 3). Among the three compounds, **3** was the most potent, inhibiting both of the cell lines at GI_50_ 0.60 and 0.38 μM, respectively. The iron-chelating activity of compounds **1**–**3**, determined by the chrome azurol S (CAS) assay [36], was IC_50_ 18, 49, and 37 μM, comparable to that of deferoxamine mesylate (IC_50_ 40 μM).

**Table 3:** Cytotoxicity data of compounds **1**–**3**.

	GI_50_ (μM)			

cell line	**1**	**2**	**3**	control^a^

3Y1 rat embryonic fibroblasts	1.4	2.8	0.60	0.058
P388 murine leukemia	1.1	11.6	0.38	0.061

^a^Doxorubicin hydrochloride.

## Conclusion

Considering the productivity of siderophores to be an essential trait for the virulence of many microbial pathogens [37], compounds **1**–**3** could also be involved in the pathogenesis of *Tenacibaculum maritimum* in fish, which is not well understood [38]. Although the genome size of *Tenacibaculum* varies from 2.5 to 7.9 Mbp, biosynthetic gene clusters for siderophores, terpenes, and non-ribosomal peptides were identified by genome mining [39], suggesting a high capability of secondary metabolism in this genus. Further investigation is underway to disclose the actual diversity of metabolites from the genus *Tenacibaculum*.

## Experimental

### General experimental procedures

UV and IR spectra were measured on a Shimadzu UV-1800 spectrophotometer and a PerkinElmer Spectrum 100 spectrophotometer, respectively. NMR spectra were recorded on a Bruker AVANCE NEO 500 spectrometer using the signals of the residual solvent protons (DMSO-*d*_6_: δ_H_ 2.50; CDCl_3_/CD_3_OD: δ_H_ 7.27) and carbons (DMSO-*d*_6_: δ_C_ 39.5; CDCl_3_/CD_3_OD: δ_C_ 77.0) as internal standards. HR–ESITOFMS spectra were measured on a Bruker compact qTOF mass spectrometer. Negative ion mode MS/MS experiments were operated on the same instrument under a multiple reaction monitoring (MRM) mode with the parameter setting “isCID = 0” and “Collision = 45”. An Agilent HP1200 HPLC system equipped with a diode array detector was used for analysis and purification. The absorbance of microtitre plate wells was read on a Thermo Scientific Multiskan Sky microplate reader.

### Microorganism

Strain C16-1 was isolated from a stony coral *Favia* sp. purchased from an aquarium vendor in Nagasaki, Japan, according to the method described previously [40]. The strain was identified as a member of the genus *Tenacibaculum* on the basis of 99.4% similarity in the 16S rRNA gene sequence (1455 nucleotides; DDBJ accession number LC498626) to *Tenacibaculum aiptasiae* a4^T^ (accession number EF416572).

### Fermentation

Strain C16-1 cultured on marine agar was inoculated into a 500 mL K-1 flask containing marine broth seed medium consisting of yeast extract (Kyokuto Pharmaceutical Industrial, Co., Ltd.) 0.2%, Tryptone (Difco Laboratories) 0.5%, dissolved in natural sea water (collected in Toyama Bay, Japan) The pH was adjusted to 7.3 before sterilization. The flasks were shaken at 30 °C for 2 days on a rotary shaker (200 rpm). The seed culture (3 mL) was transferred into 30 500 mL K-1 flasks each containing 100 mL of A11M production medium (pH 7.0) consisting of 2.5% soluble starch, 0.2% glucose, 0.5% yeast extract, 0.5% Hipolypeptone (Wako Pure Chemical Industries, Ltd), NZ amine (Wako Pure Chemical Industries, Ltd), CaCO_3_ 0.3%, and 1% Diaion HP-20 (Mitsubishi Chemical Co.) in natural sea water. The inoculated flasks were placed on a rotary shaker (200 rpm) at 30 °C for 7 days.

### Extraction and isolation

At the end of the fermentation period, 100 mL of 1-butanol were added to each flask and the flasks were shaken for 1 h. The mixture was centrifuged at 6000 rpm for 10 min and the organic layer was separated from the aqueous layer containing the mycelium. Evaporation of the solvent gave 6.54 g of extract from 3 L of culture. The extract (6.54 g) was subjected to silica gel column chromatography with a step gradient of CHCl_3_/MeOH 1:0, 20:1, 10:1, 4:1, 2:1, 1:1, and 0:1 (v/v). Fraction 4 (4:1) was concentrated to provide 2.46 g of a brown solid, which was further purified by ODS column chromatography with a gradient of MeCN/0.1% HCO_2_H 2:8, 3:7, 4:6, 5:5, 6:4, 7:3, and 8:2 (v/v). Fraction 5 (7:3) was concentrated to dryness and the residual solid (527 mg) was applied to the preparative HPLC (Cosmosil Cholester Packed Column, 10 × 250 mm, Nacalai Tesque) using an isocratic elution with 50% MeCN in 0.1% HCO_2_H over 40 min at a flow rate of 4 mL/min, yielding tenacibactin K (**1**, 31.6 mg, *t*_R_ 28.0 min), tenacibactin L (**2**, 2.8 mg, *t*_R_ 22.0 min), and tenacibactin M (**3**, 18.2 mg, *t*_R_ 34.4 min).

Tenacibactin K (**1**): pale brown powder; UV (MeOH) λ_max_ nm (log ε): 201 (4.82) nm; IR (ATR) ν_max_: 3305, 2916, 2849, 1613, 1538, 1466 cm^−1^; ^1^H and ^13^C NMR, Table 1; HR–ESITOFMS (*m*/*z*): [M − H]^−^ calcd for C_33_H_60_N_5_O_8_, 654.4447; found, 654.4449; [M + Na]^+^ calcd for C_33_H_61_N_5_O_8_Na, 678.4412; found, 678.4412.

Tenacibactin L (**2**): pale brown powder; UV (MeOH) λ_max_ nm (log ε): 202 (4.21) nm; IR (ATR) ν_max_: 3306, 2916, 2849, 1613, 1538, 1466 cm^−1^; ^1^H and ^13^C NMR, Table 2; HR–ESITOFMS (*m*/*z*): [M − H]^−^ calcd for C_33_H_60_N_5_O_8_, 654.4447; found, 654.4445; [M + Na]^+^ calcd for C_33_H_61_N_5_O_8_Na, 678.4412; found, 678.4410.

Tenacibactin M (**3**): pale brown powder; UV (MeOH) λ_max_ nm (log ε): 202 (4.35) nm; IR (ATR) ν_max_: 3306, 2916, 2849, 1613, 1538, 1466 cm^−1^; ^1^H and ^13^C NMR, Table 2; HR–ESITOFMS (*m*/*z*): [M − H]^−^ calcd for C_33_H_62_N_5_O_8_, 6546.4604; found, 656.4604; [M + Na]^+^ calcd for C_33_H_63_N_5_O_8_Na, 680.4569; found, 680.4567.

### Bioassays

Antimicrobial activity was examined as previously reported [41]. *Kocuria rhizophila* ATCC9341, *Staphylococcus aureus* FDA209P JC-1, *Ralstonia solanacearum* SUPP1541, *Escherichia coli* NIHJ JC-2, *Rhizobium radiobacter* NBRC14554, and *Candida albicans* NBRC0197 were used as indication strains. Cytotoxicity against 3Y1 rat embryonic fibroblasts and P388 murine leukemia cells were evaluated according to the protocols described in references [40–41].

### CAS assay

Compounds **1**–**3**, along with deferoxamine mesylate as a reference, were serially half-diluted in a 96-well round-bottomed microtitre plate. To each well were added 100 µL of CAS-Fe^3+^ solution [36]. The volumes of the vehicle solvents, DMSO for **1**–**3** and distilled water for deferoxamine mesylate, were reduced to 5% at maximum of the final test solution. After shaking the plate gently for 4 h at 25 °C, the remaining CAS-Fe^3+^ complex in each well was quantified by measuring the absorbance at 630 nm by a microplate reader. The results were translated into ratios of Fe^3+^-complexed dye at each concentration, which were plotted on single-logarithmic charts to deduce IC_50_ values. The tests were run in triplicate for compounds **1**, **3**, and deferoxamine mesylate while only a single set experiment was possible for **2** due to its limited availability.

## Supporting Information

File 1 Copies of UV, IR, MS/MS, and NMR spectra for compounds **1**–**3**.
