# Supplementary material for: Tenacibactins K–M, cytotoxic siderophores from a coral-associated gliding bacterium of the genus Tenacibaculum
Source: Beilstein J Org Chem. 2022 Jan 13;18:110–9. doi: 10.3762/bjoc.18.12 (PMC8767563; doi:10.3762/bjoc.18.12)
Supplement: File 1 — Copies of UV, IR, MS/MS, and NMR spectra for compounds 1–3. [file Beilstein_J_Org_Chem-18-110-s001.pdf]

BEILSTEIN JOURNAL OF ORGANIC CHEMISTRY

Supporting Information

for

Tenacibactins K–M, cytotoxic siderophores from a coral-associated gliding bacterium of the genus *Tenacibaculum*

Yasuhiro Igarashi, Yiwei Ge, Tao Zhou, Amit Raj Sharma, Enjuro Harunari, Naoya Oku and Agus Trianto

Beilstein J. Org. Chem. **2022,** *18,* 110–119. doi:10.3762/bjoc.18.12

Copies of UV, IR, MS/MS, and NMR spectra for compounds 1–3

License and Terms: This is a supporting information file under the terms of the Creative Commons Attribution License (https://creativecommons.org/licenses/by/4.0). Please note that the reuse, redistribution and reproduction in particular requires that the author(s) and source are credited and that individual graphics may be subject to special legal provisions.
The license is subject to the *Beilstein Journal of Organic Chemistry* terms and conditions: (https://www.beilstein-journals.org/bjoc/terms)

Table of contents

Figure S1: UV spectrum of tenacibactin K (**1**).
Figure S2: IR spectrum of **1**.
Figure S3: ^{1}H NMR spectrum of **1** (500 MHz, DMSO-d_6, 25 °C).
Figure S4: ^{13}C NMR spectrum of **1** (125 MHz, DMSO-d_6, 25 °C).
Figure S5: COSY spectrum of **1** (500 MHz, DMSO-d_6, 25 °C).
Figure S6: HSQC spectrum of **1** (500 MHz, DMSO-d_6, 25 °C).
Figure S7: HMBC spectrum of **1** (500 MHz, DMSO-d_6, 25 °C).
Figure S8: ROESY spectrum of **1** (500 MHz, DMSO-d_6, 25 °C).
Figure S9: ^{1}H NMR spectrum of **1** (500 MHz, $CDCl_3/CD_3OD$ 3:7, 25 °C).
Figure S10: ^{13}C NMR spectrum of **1** (125 MHz, $CDCl_3/CD_3OD$ 3:7, 25 °C).
Figure S11: HMBC spectrum of **1** (500 MHz, $CDCl_3/CD_3OD$ 3:7, 25 °C).
Figure S12: UV spectrum of tenacibactin L (**2**).
Figure S13: IR spectrum of **2**.
Figure S14: ^{1}H NMR spectrum of **2** (500 MHz, DMSO-d_6, 25 °C).
Figure S15: ^{13}C NMR spectrum of **2** (125 MHz, DMSO-d_6, 25 °C).
Figure S16: COSY spectrum of **2** (500 MHz, DMSO-d_6, 25 °C).
Figure S17: HSQC spectrum of **2** (500 MHz, DMSO-d_6, 25 °C).
Figure S18: HMBC spectrum of **2** (500 MHz, DMSO-d_6, 25 °C).
Figure S19: MS/MS spectrum of **2**.
Scheme S20: Fragmentation pathway for **2**.
Figure S21: UV spectrum of tenacibactin M (**3**).
Figure S22: IR spectrum of **3**.
Figure S23: ^{1}H NMR spectrum of **3** (500 MHz, DMSO-d_6, 25 °C).
Figure S24: ^{13}C NMR spectrum of **3** (125 MHz, DMSO-d_6, 25 °C).
Figure S25: COSY spectrum of **3** (500 MHz, DMSO-d_6, 25 °C).
Figure S26: HSQC spectrum of **3** (500 MHz, DMSO-d_6, 25 °C).
Figure S27: HMBC spectrum of **3** (500 MHz, DMSO-d_6, 25 °C).
Figure S28: MS/MS spectrum of **3**.
Scheme S29: Fragmentation pathway for **3**.

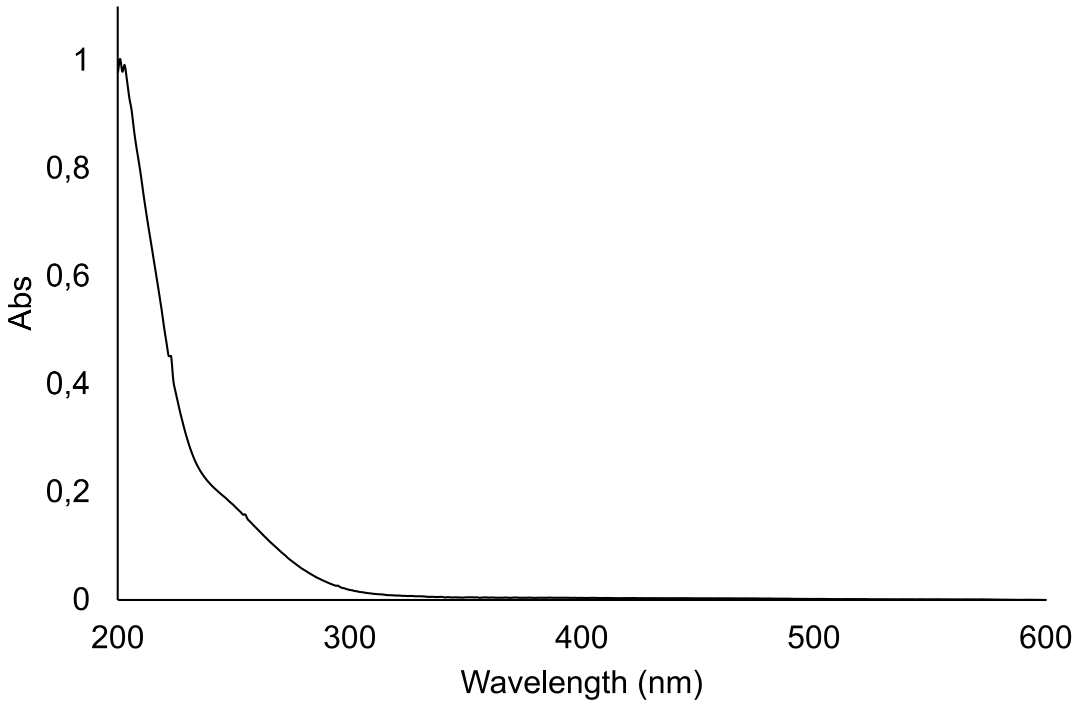

Figure S1: UV spectrum of tenacibactin K (**1**).

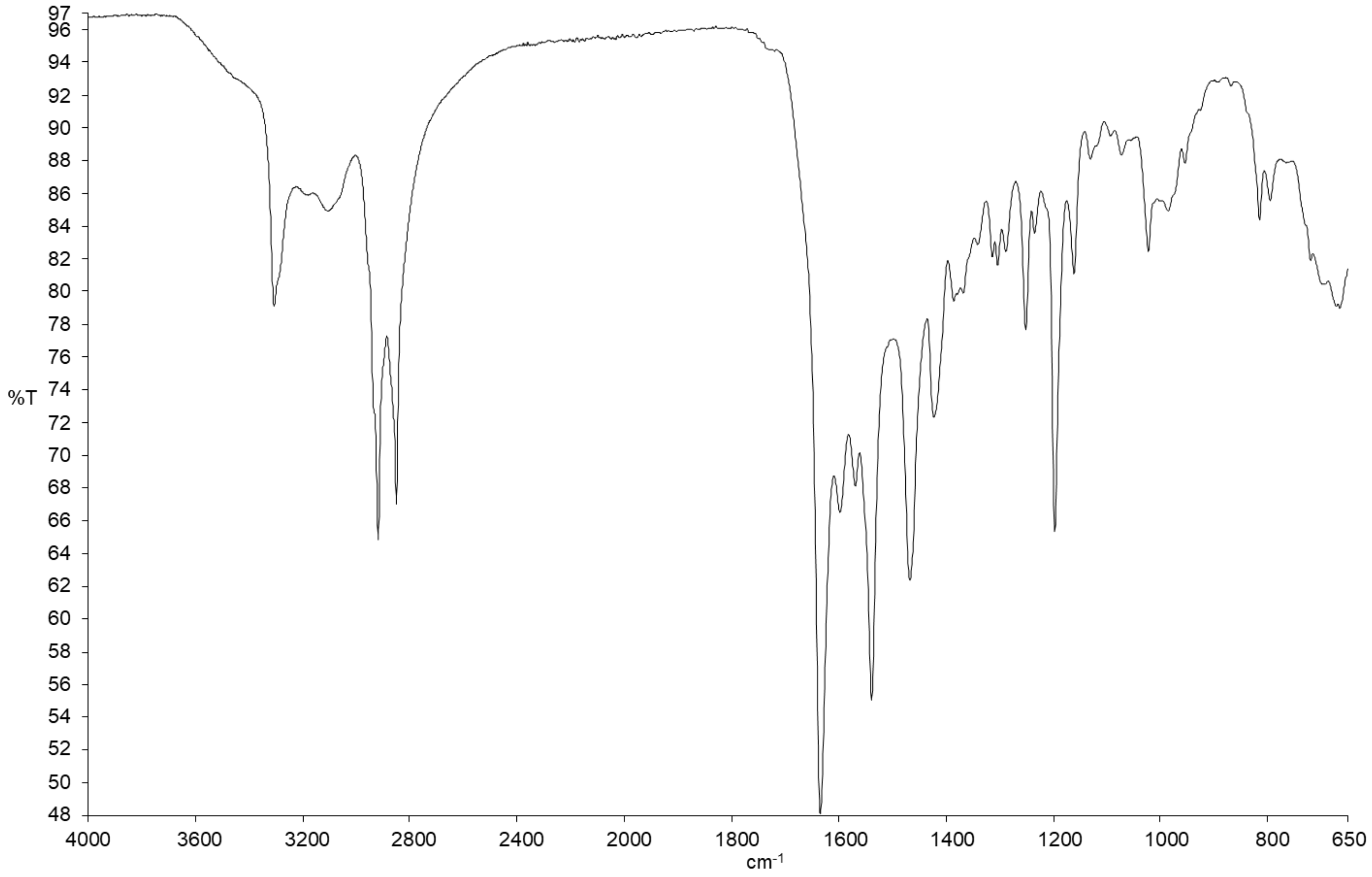

Figure S2: IR spectrum of **1**.

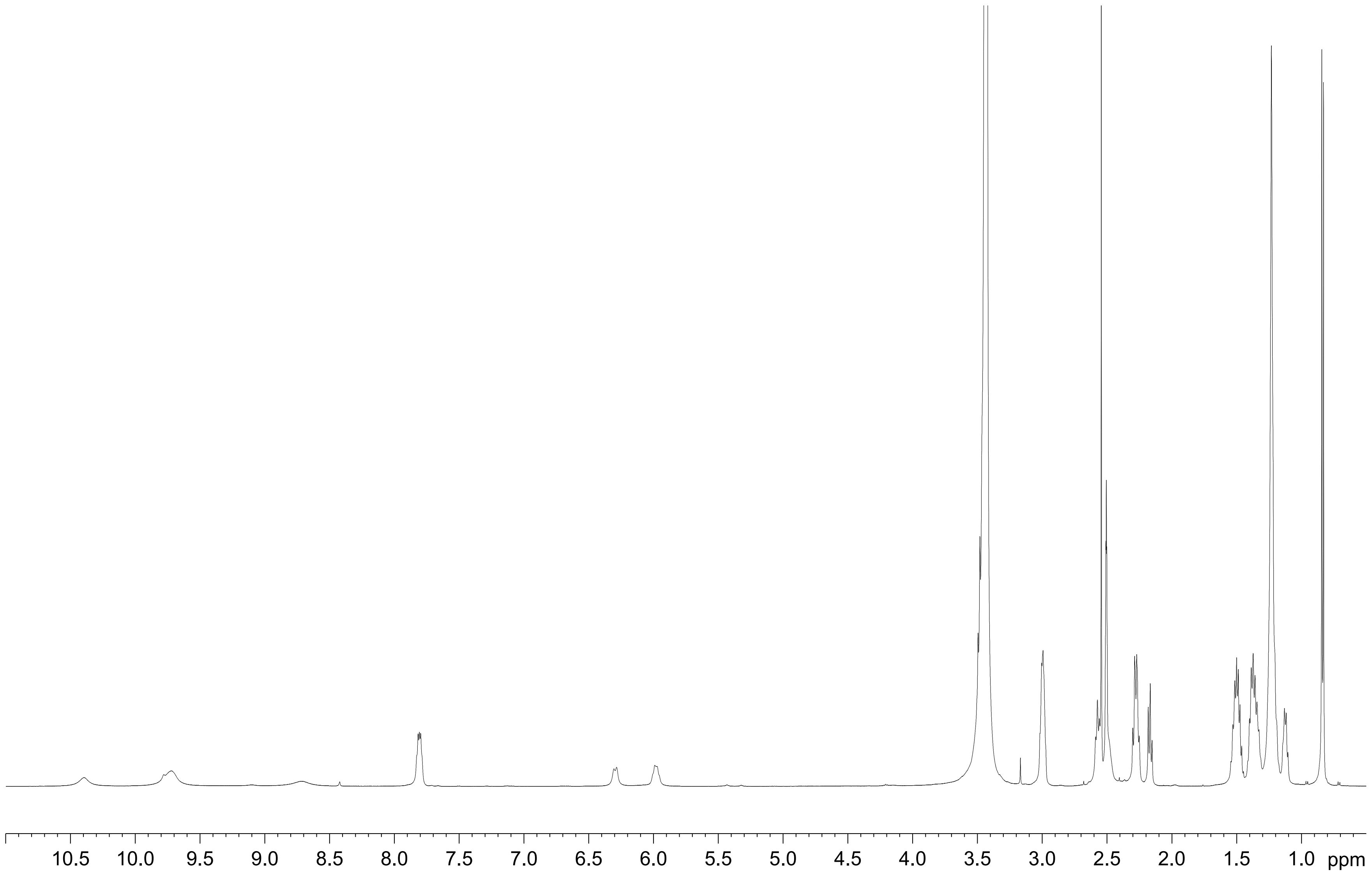

Figure S3: ^{1}H NMR spectrum of **1** (500 MHz, DMSO-d_6, 25 °C).

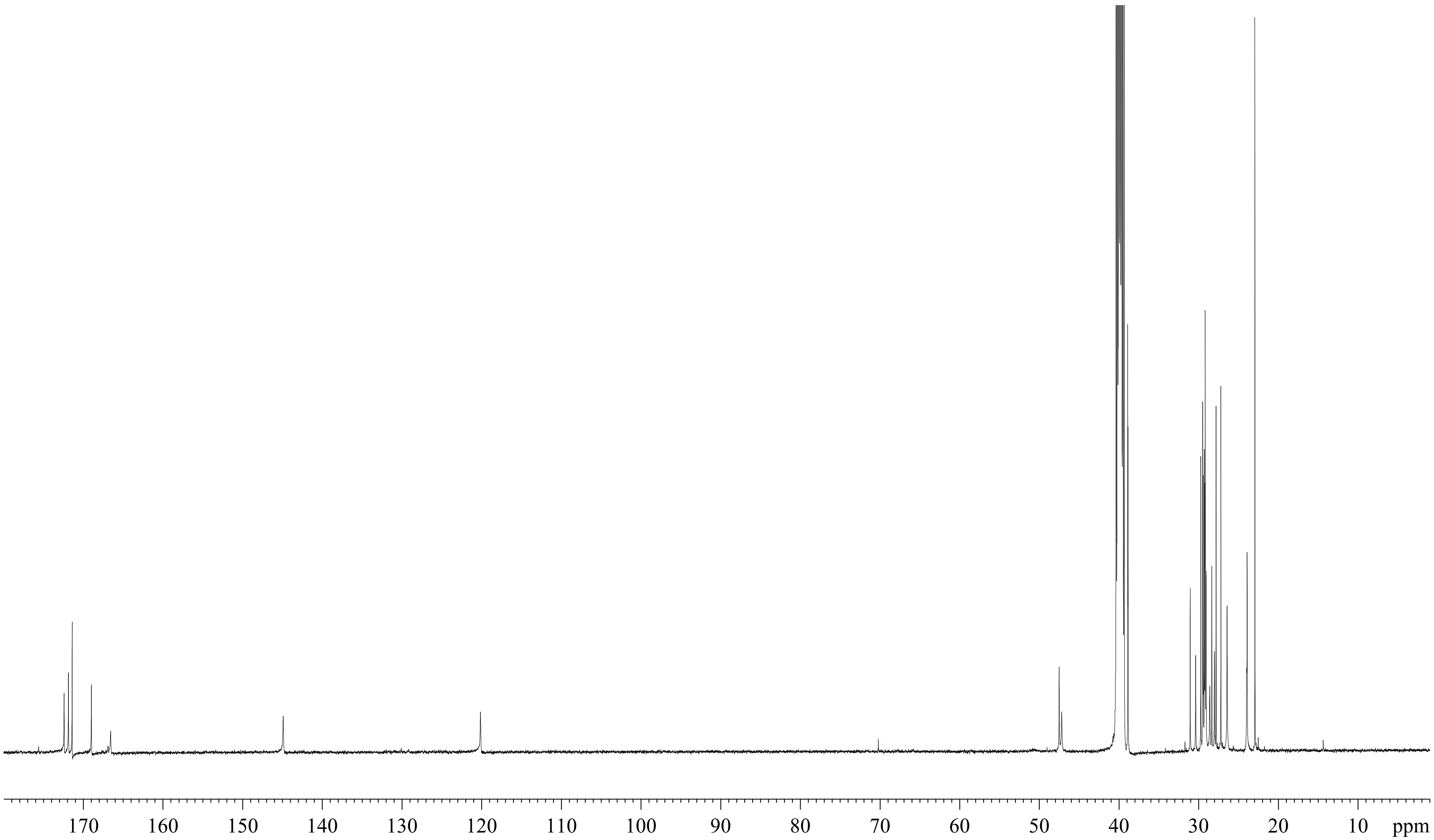

Figure S4: ^{13}C NMR spectrum of **1** (500 MHz, DMSO-d_6, 25 °C).

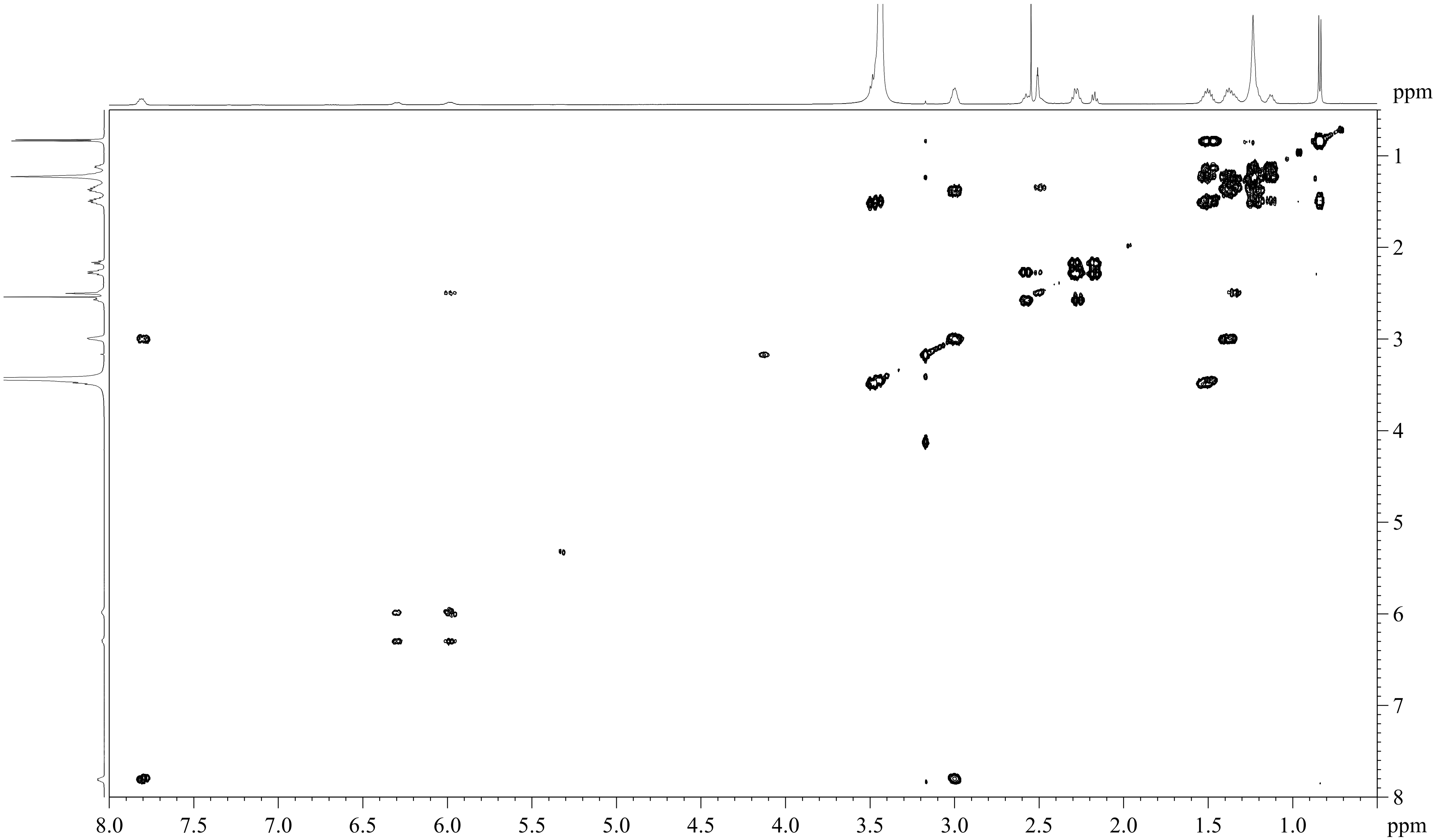

Figure S5: COSY spectrum of **1** (500 MHz, DMSO-d_6, 25 °C).

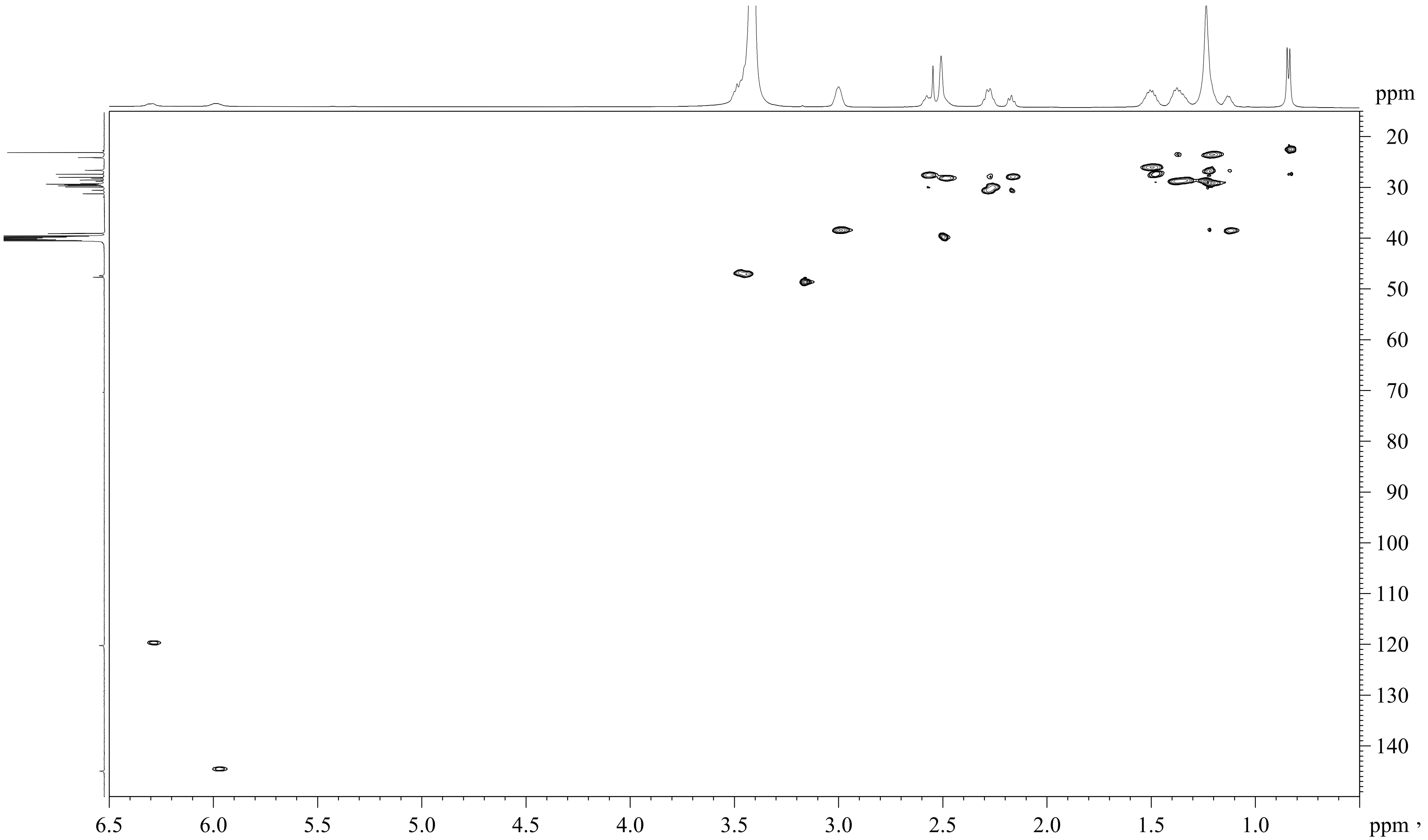

Figure S6: HSQC spectrum of **1** (500 MHz, DMSO-d_6, 25 °C).

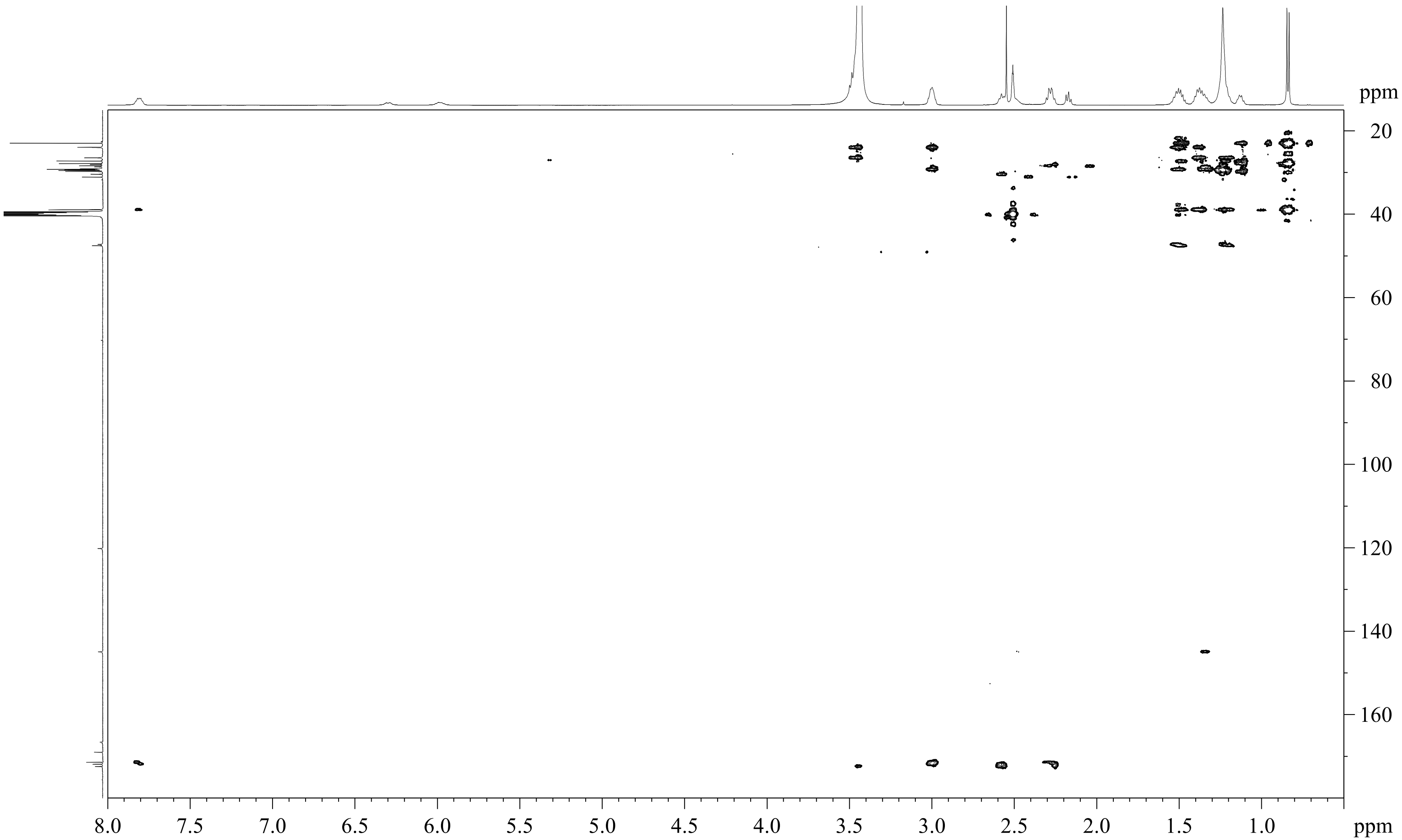

Figure S7: HMBC spectrum of **1** (500 MHz, DMSO-d_6, 25 °C).

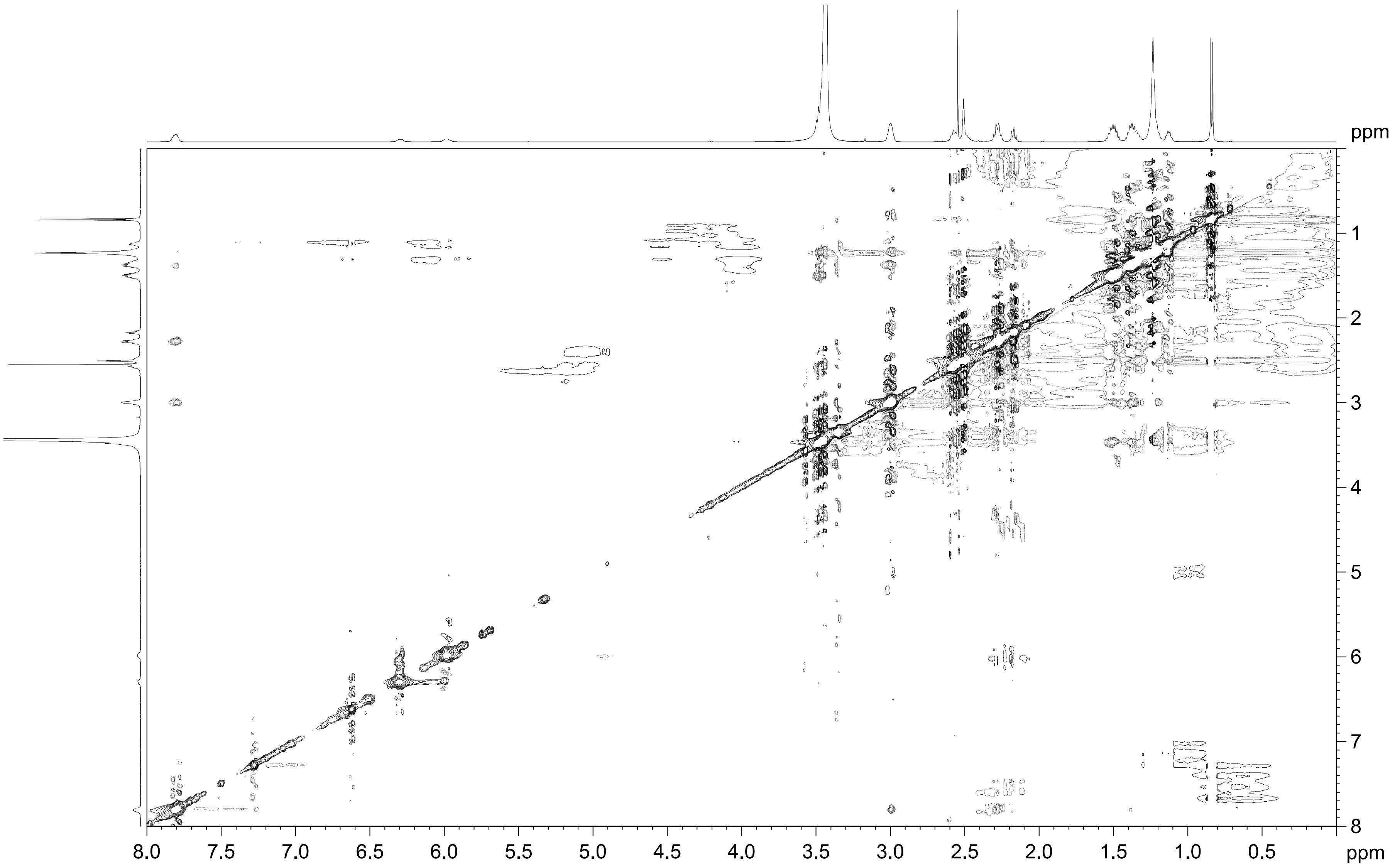

Figure S8: ROESY spectrum of **1** (500 MHz, DMSO-d_6, 25 °C).

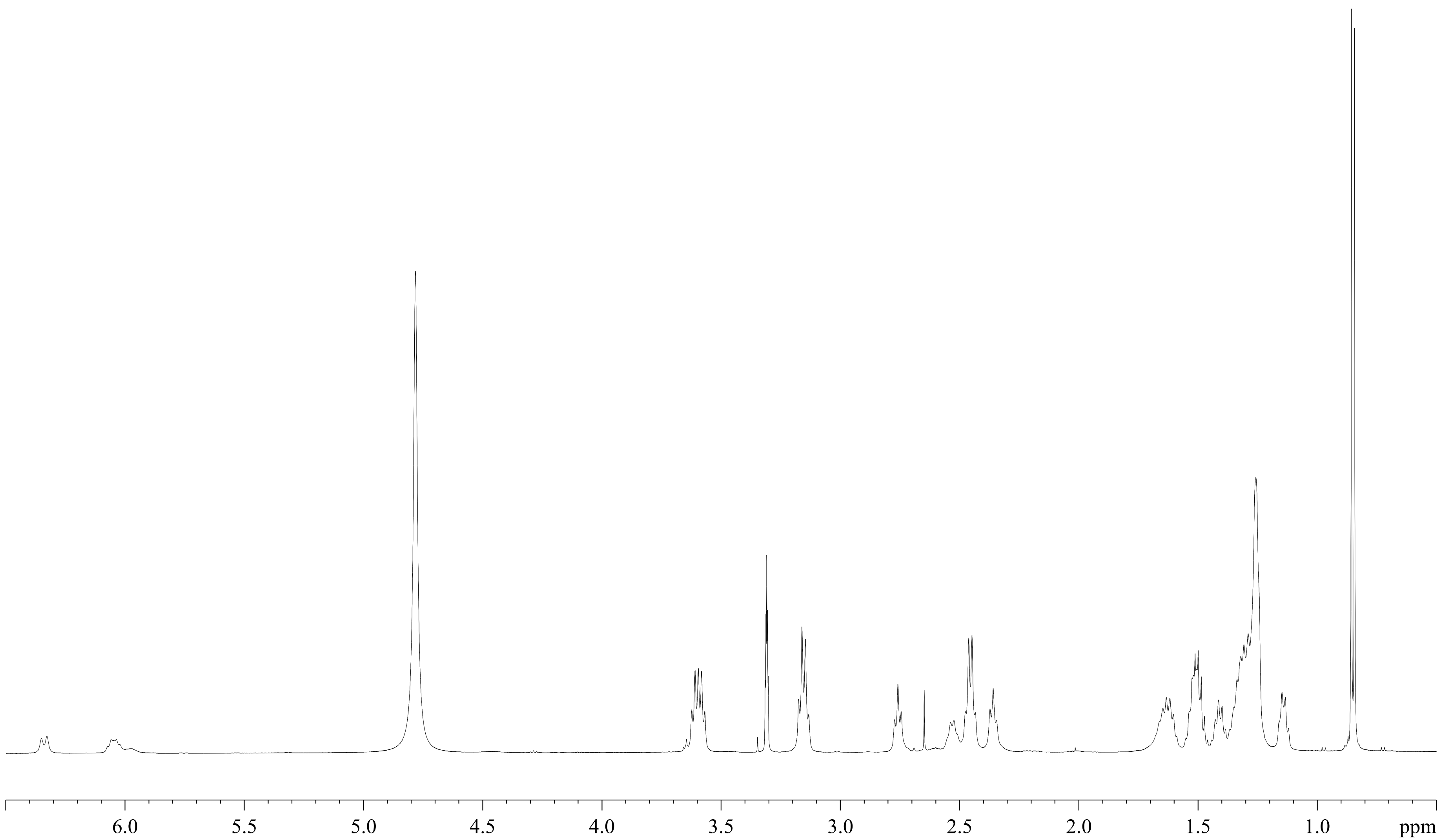

Figure S9: ^{1}H NMR spectrum of **1** (500 MHz, $CDCl_3/CD_3OD$ 3:7, 25 °C).

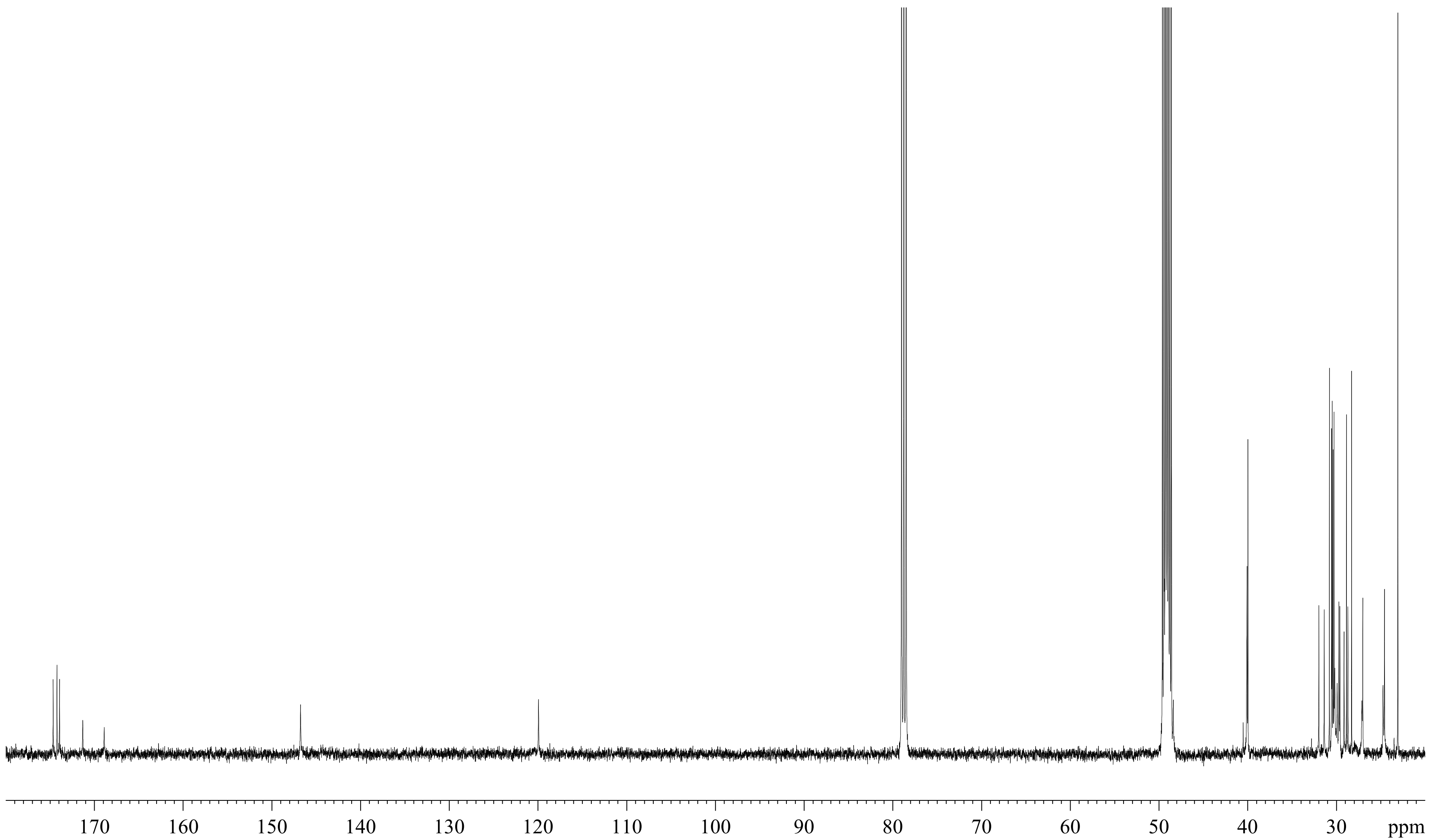

Figure S10: ^{13}C NMR spectrum of **1** (125 MHz, $CDCl_3/CD_3OD$ 3:7, 25 °C).

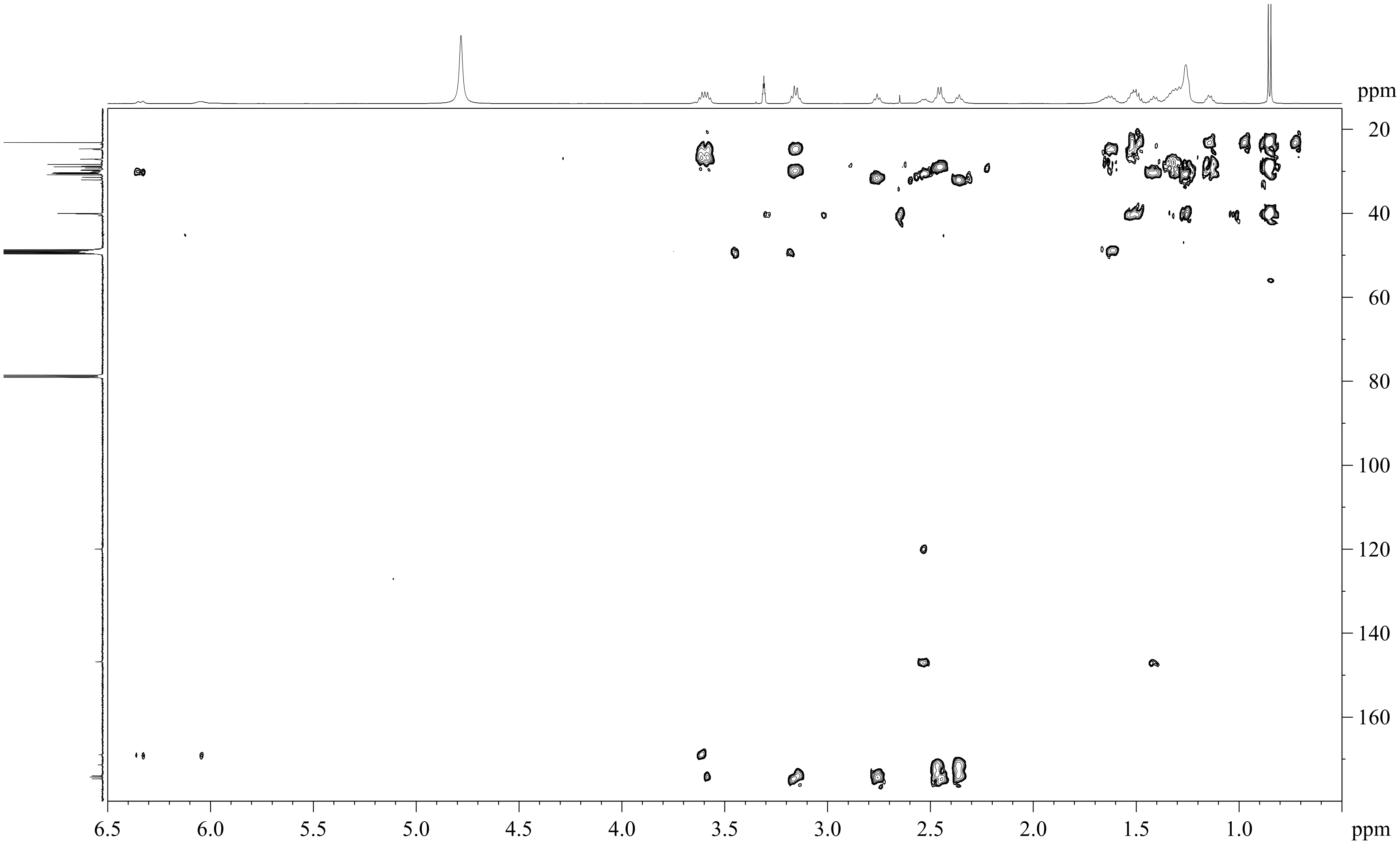

Figure S11: HMBC spectrum of **1** (500 MHz, $CDCl_3/CD_3OD$ 3:7, 25 °C).

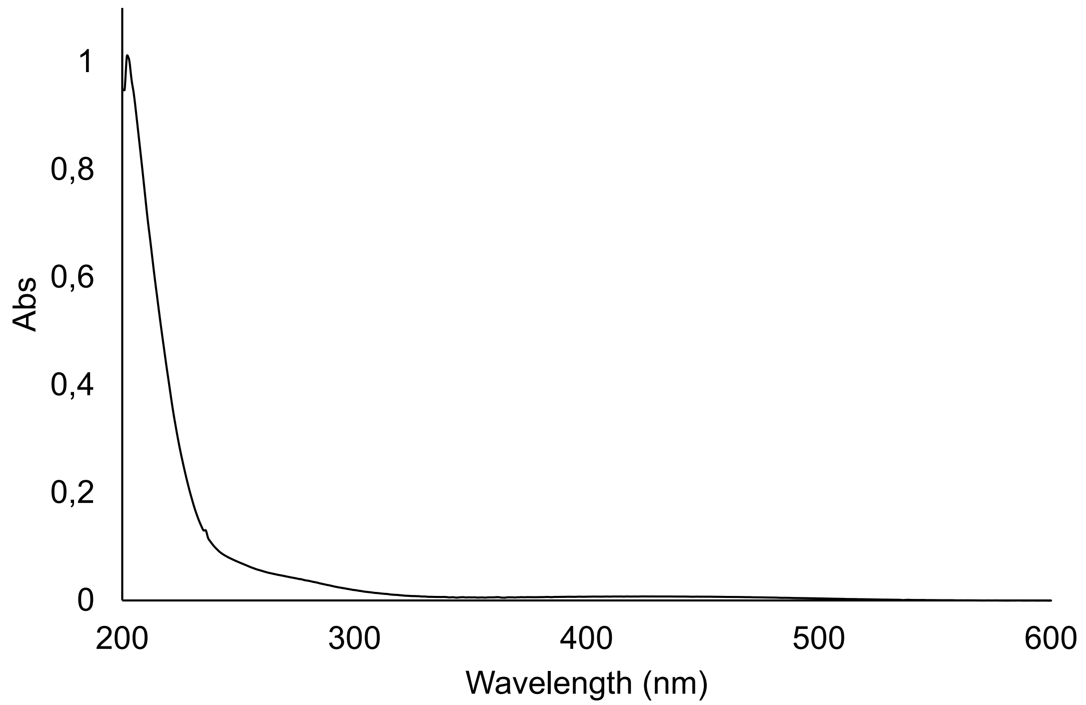

Figure S12: UV spectrum of tenacibactin L (**2**).

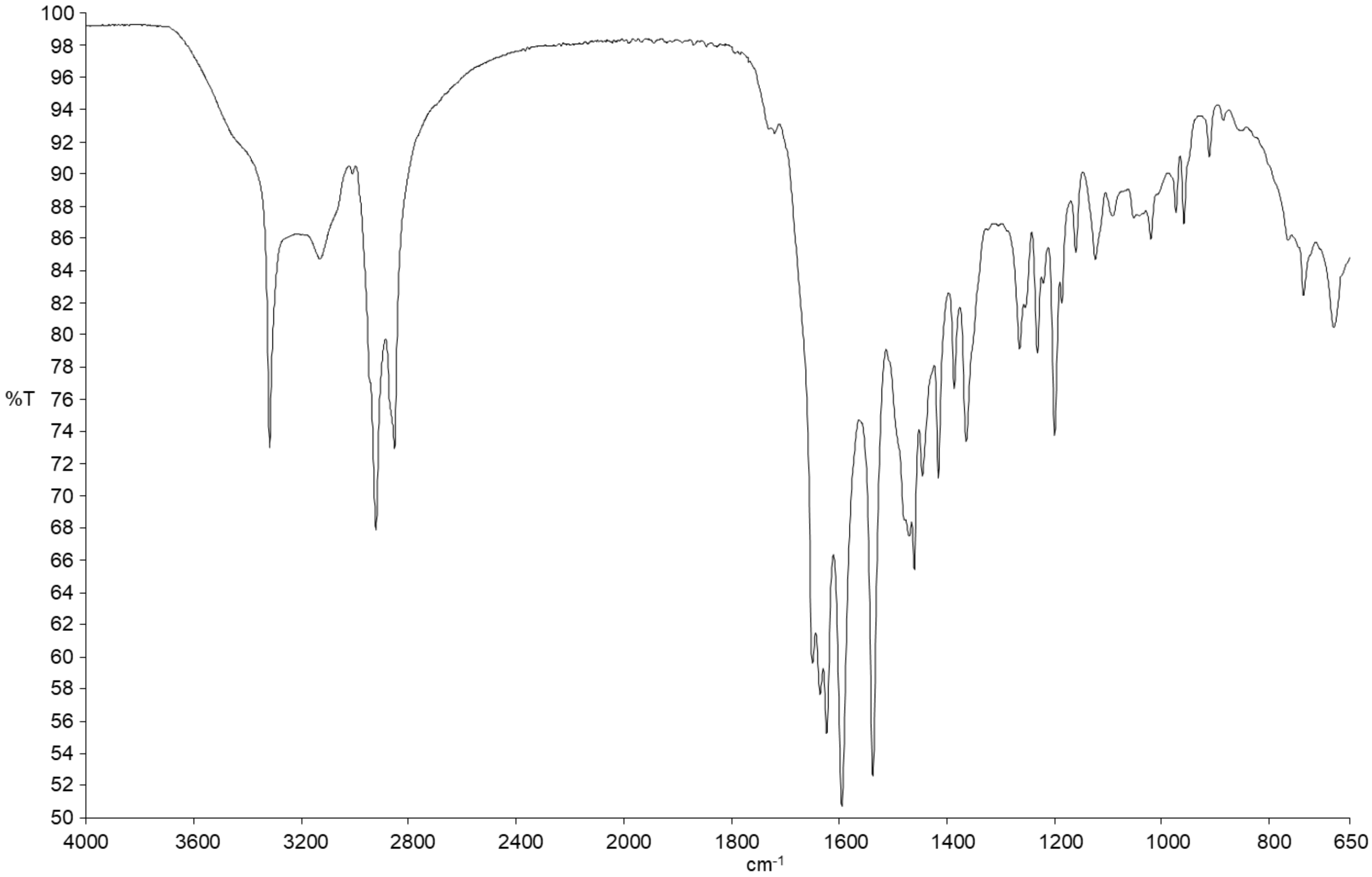

Figure S13: IR spectrum of **2**.

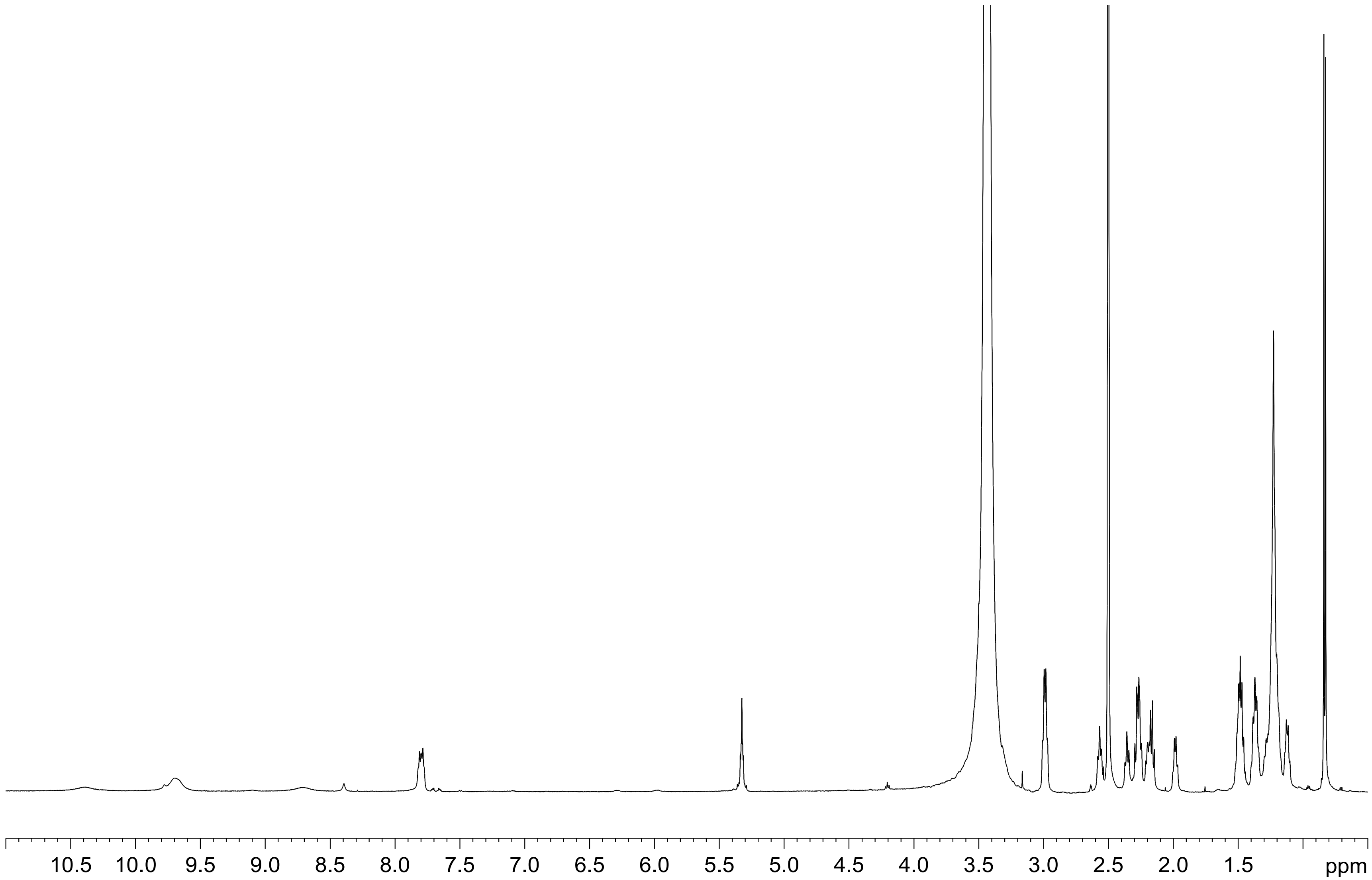

Figure S14: ^{1}H NMR spectrum of **2** (500 MHz, DMSO-d_6, 25 °C).

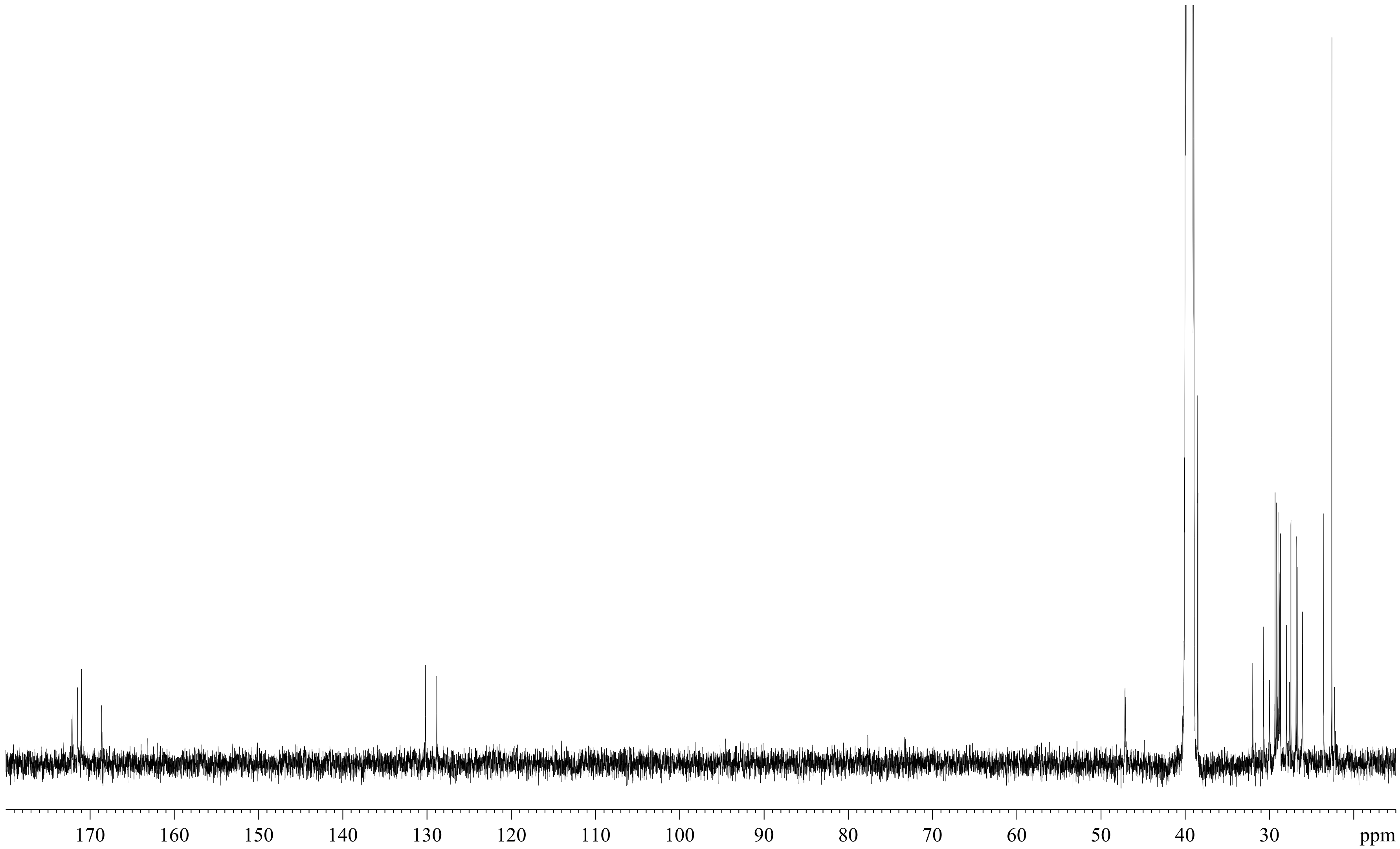

Figure S15: ^{13}C NMR spectrum of **2** (125 MHz, DMSO-d_6, 25 °C).

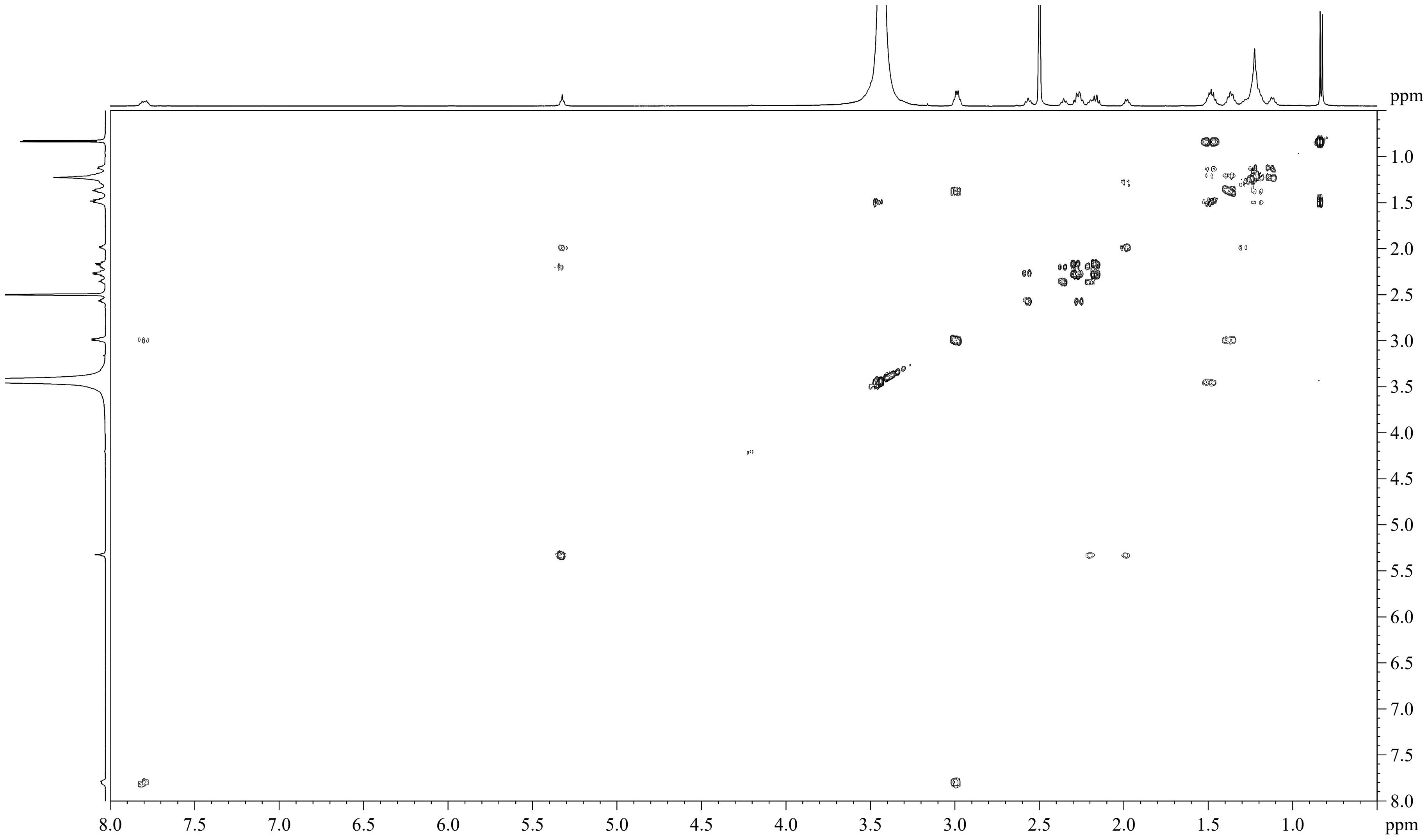

Figure S16: COSY spectrum of **2** (500 MHz, DMSO-d_6, 25 °C).

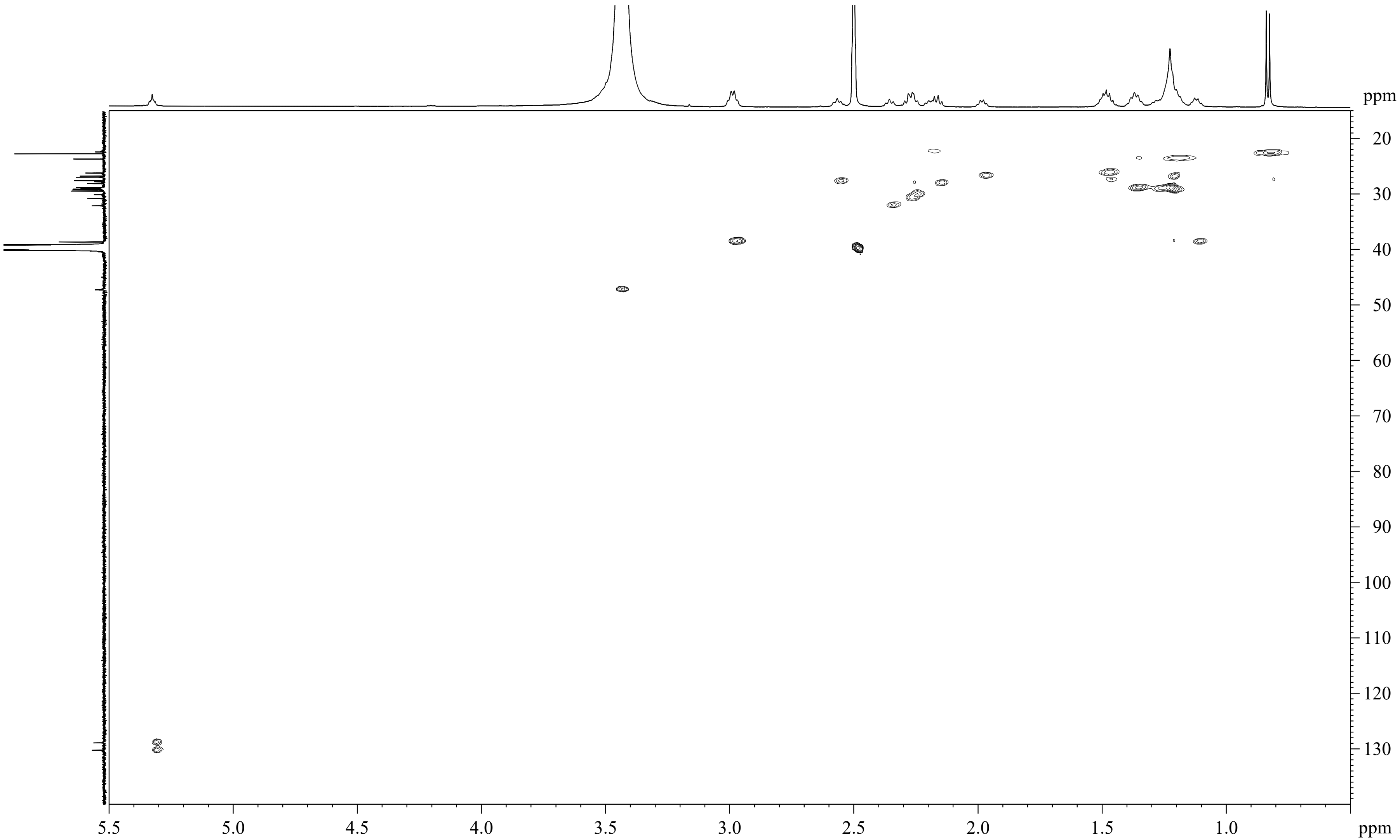

Figure S17: HSQC spectrum of **2** (500 MHz, DMSO-d_6, 25 °C).

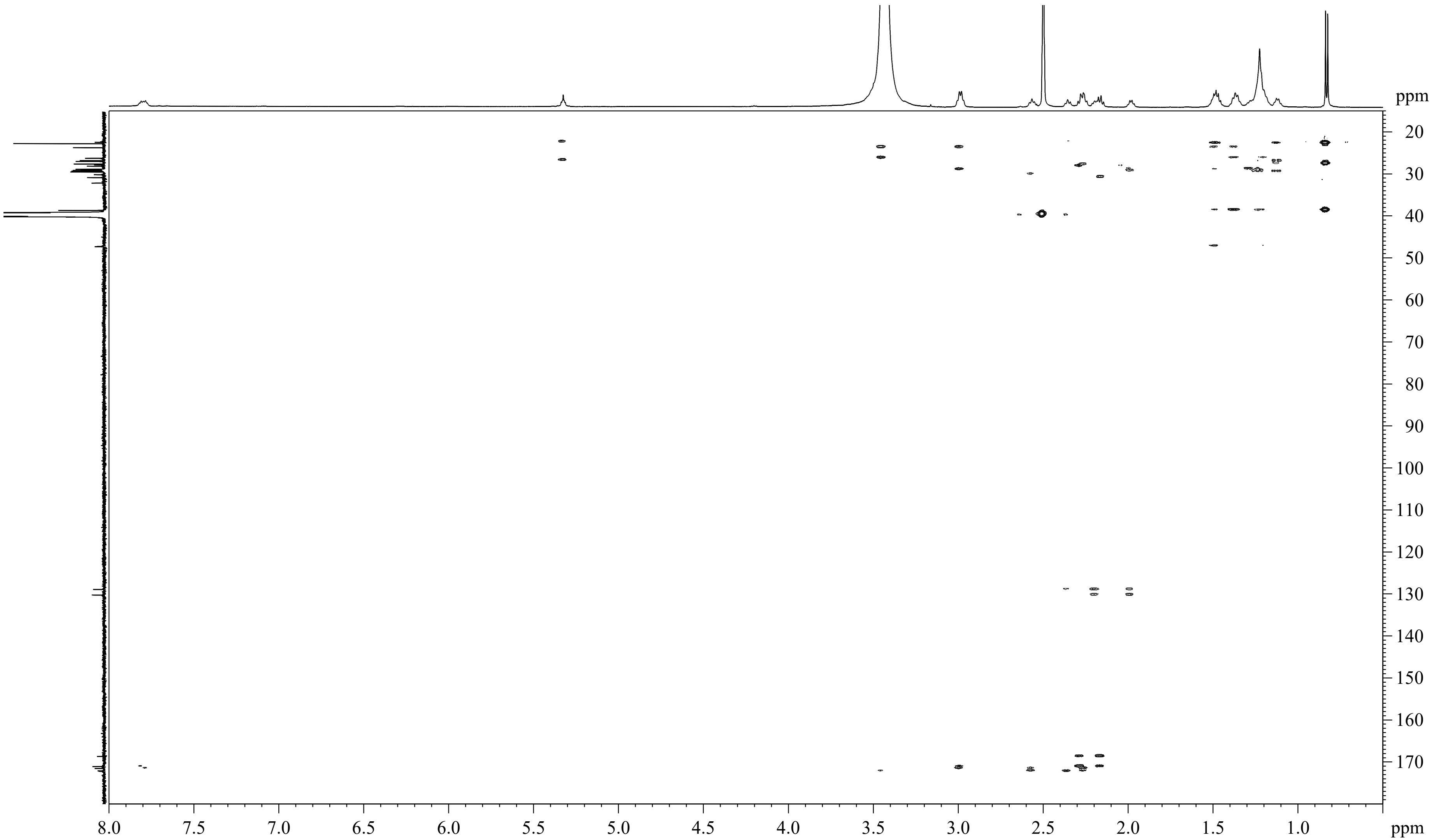

Figure S18: HMBC spectrum of **2** (500 MHz, DMSO-d_6, 25 °C).

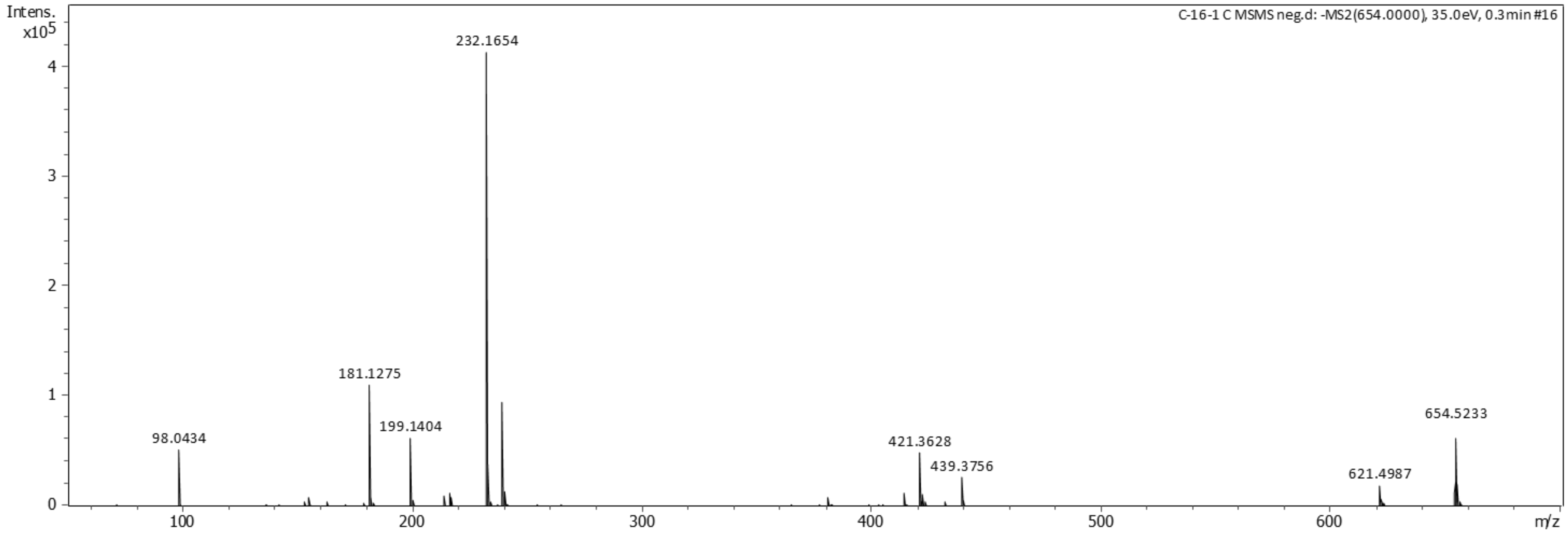

Figure S19: MS/MS spectrum of **2**.

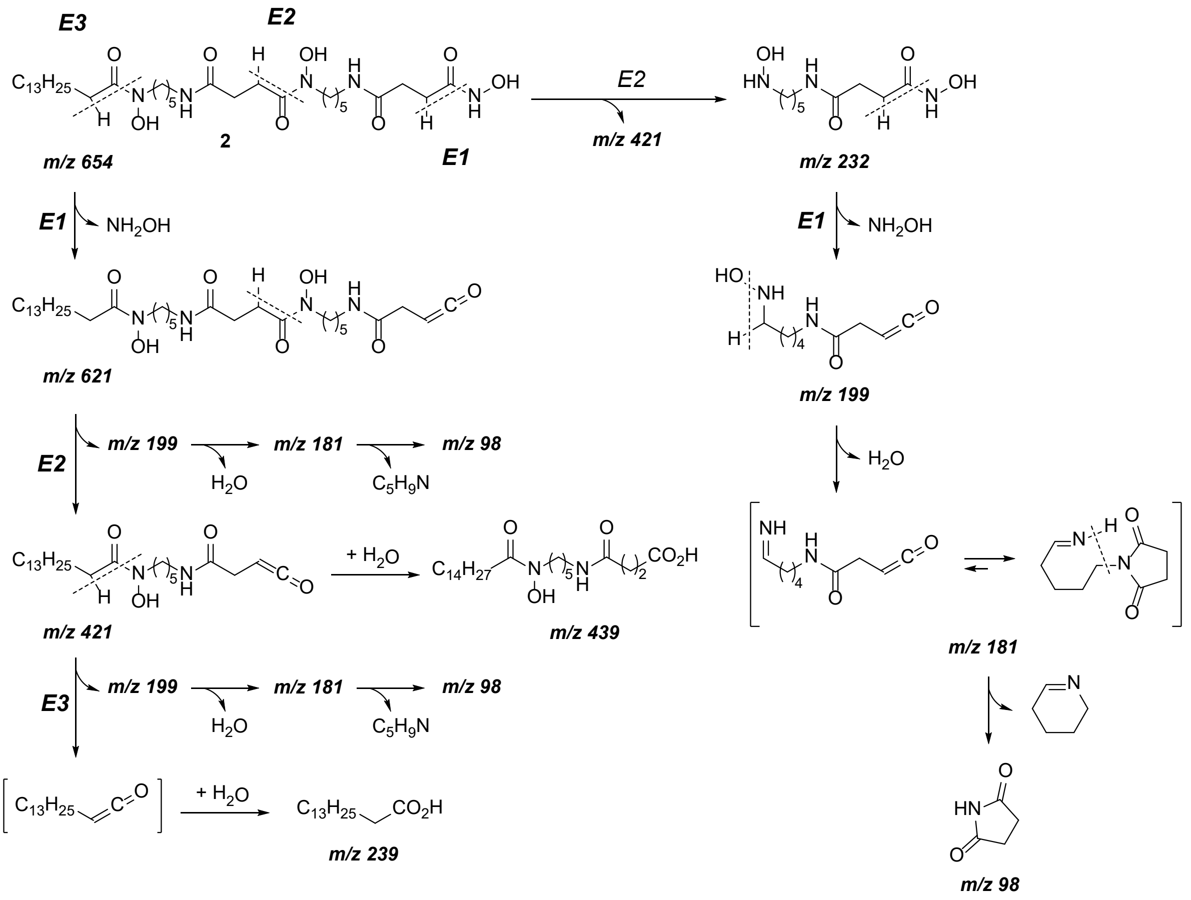

Scheme S20: Fragmentation pathway for **2**.

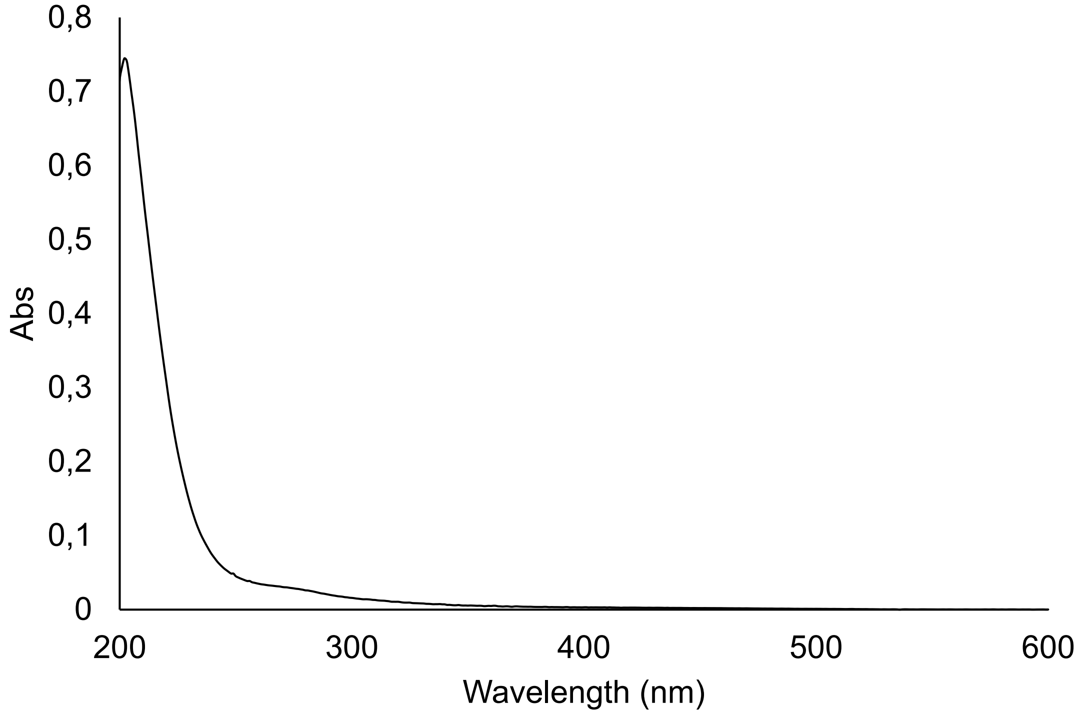

Figure S21: UV spectrum of tenacibactin M (**3**).

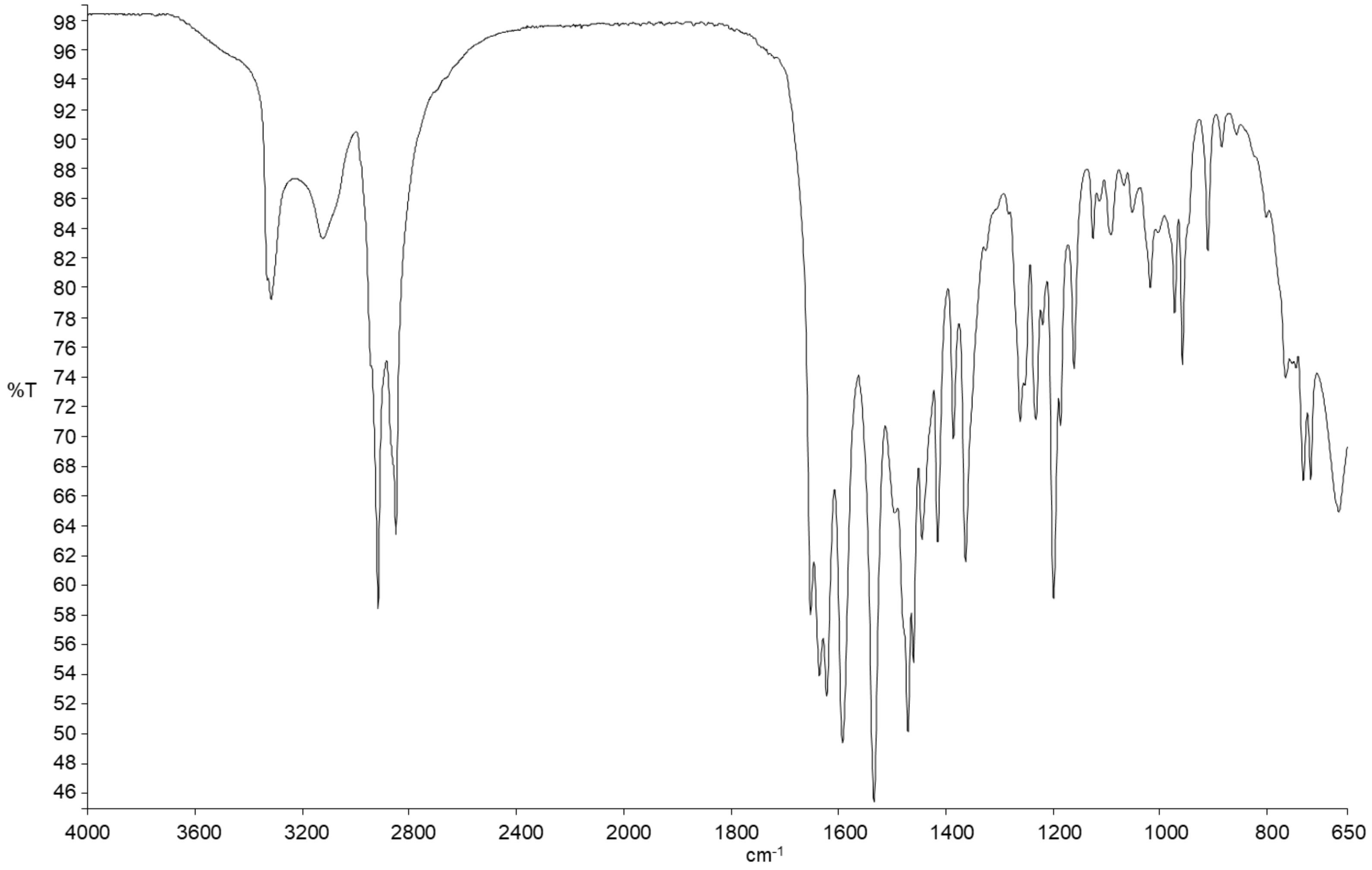

Figure S22: IR spectrum of **3**.

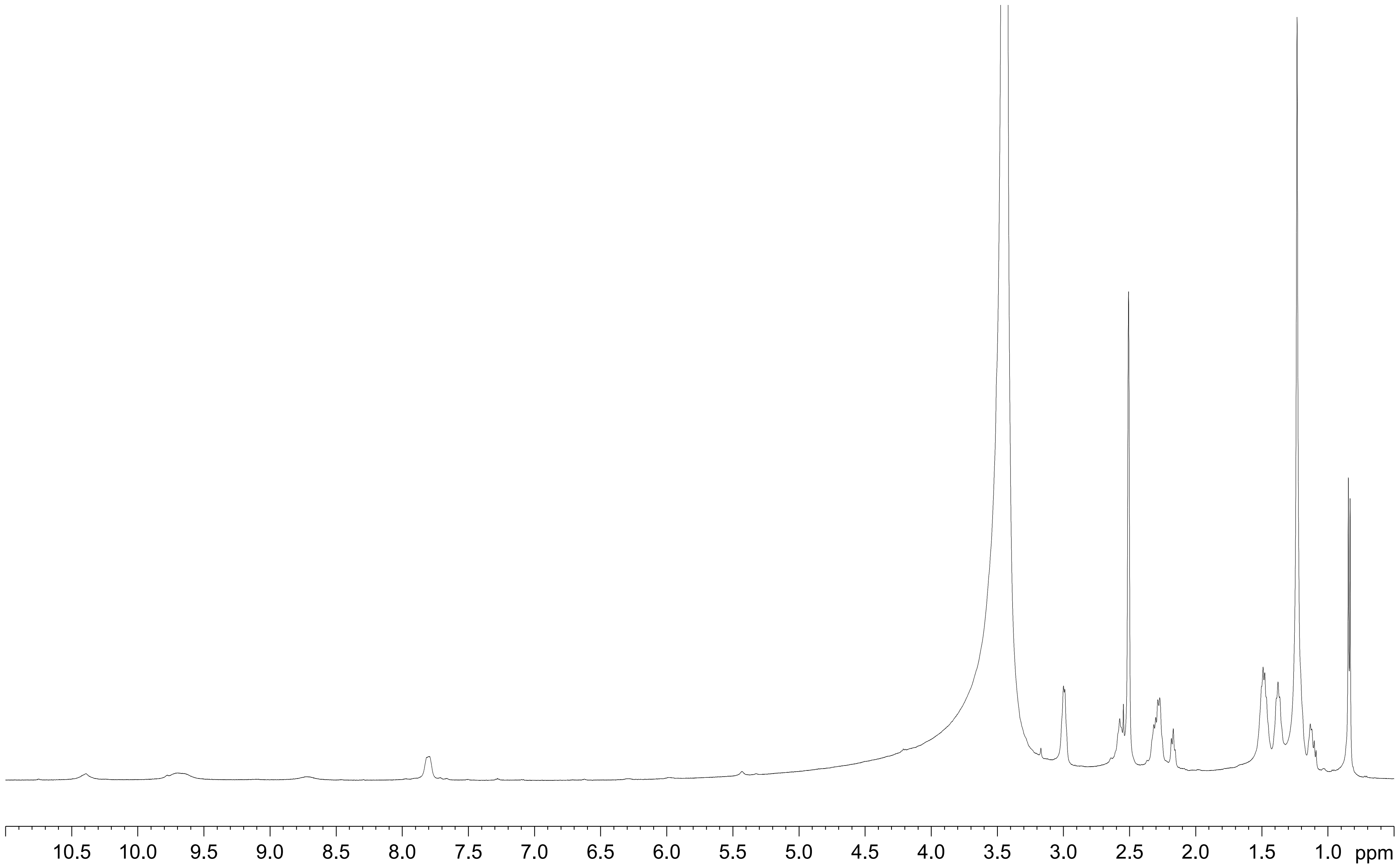

Figure S23: ^{1}H NMR spectrum of **3** (500 MHz, DMSO-d_6, 25 °C).

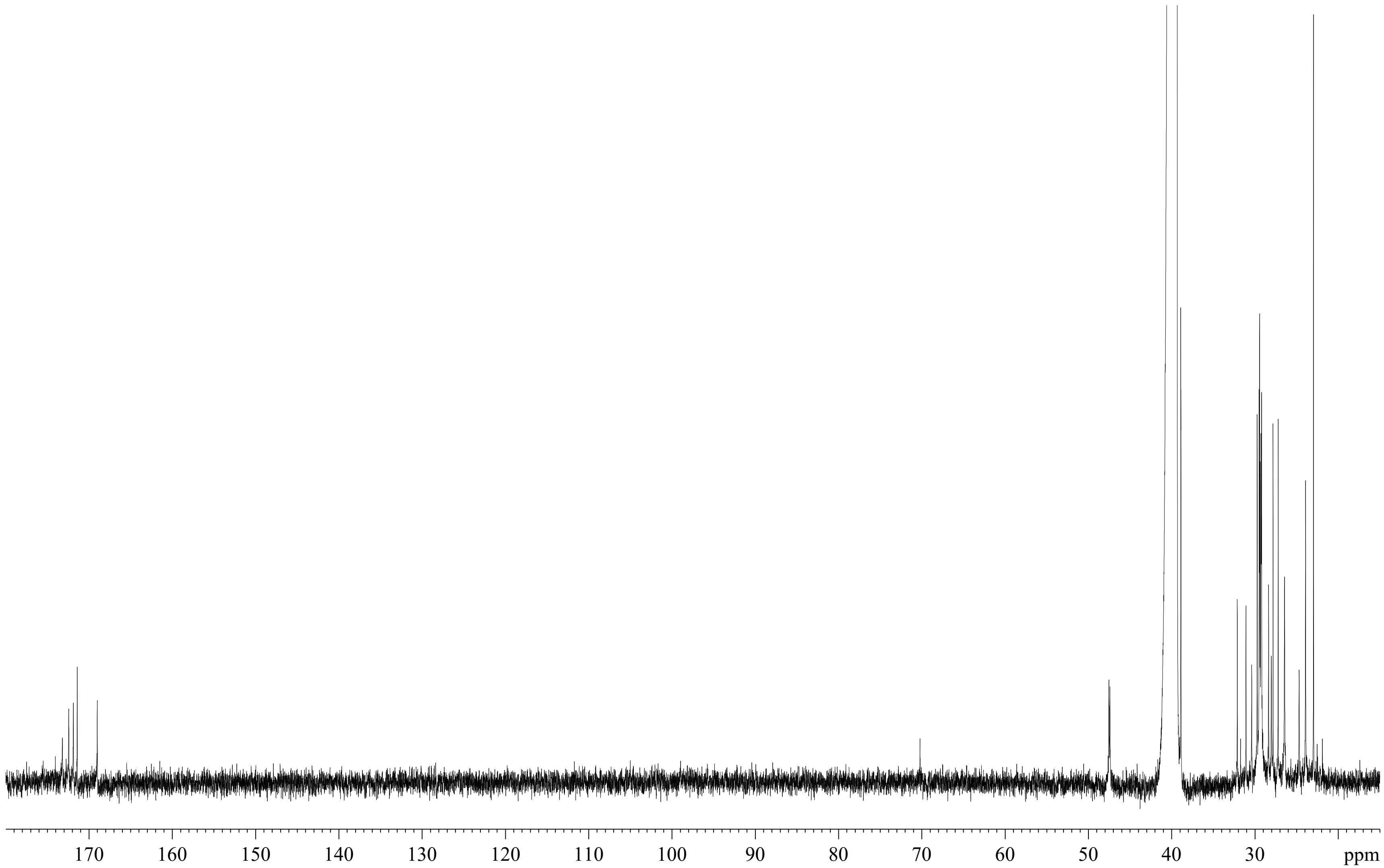

Figure S24: ^{13}C NMR spectrum of **3** (125 MHz, DMSO-d_6, 25 °C).

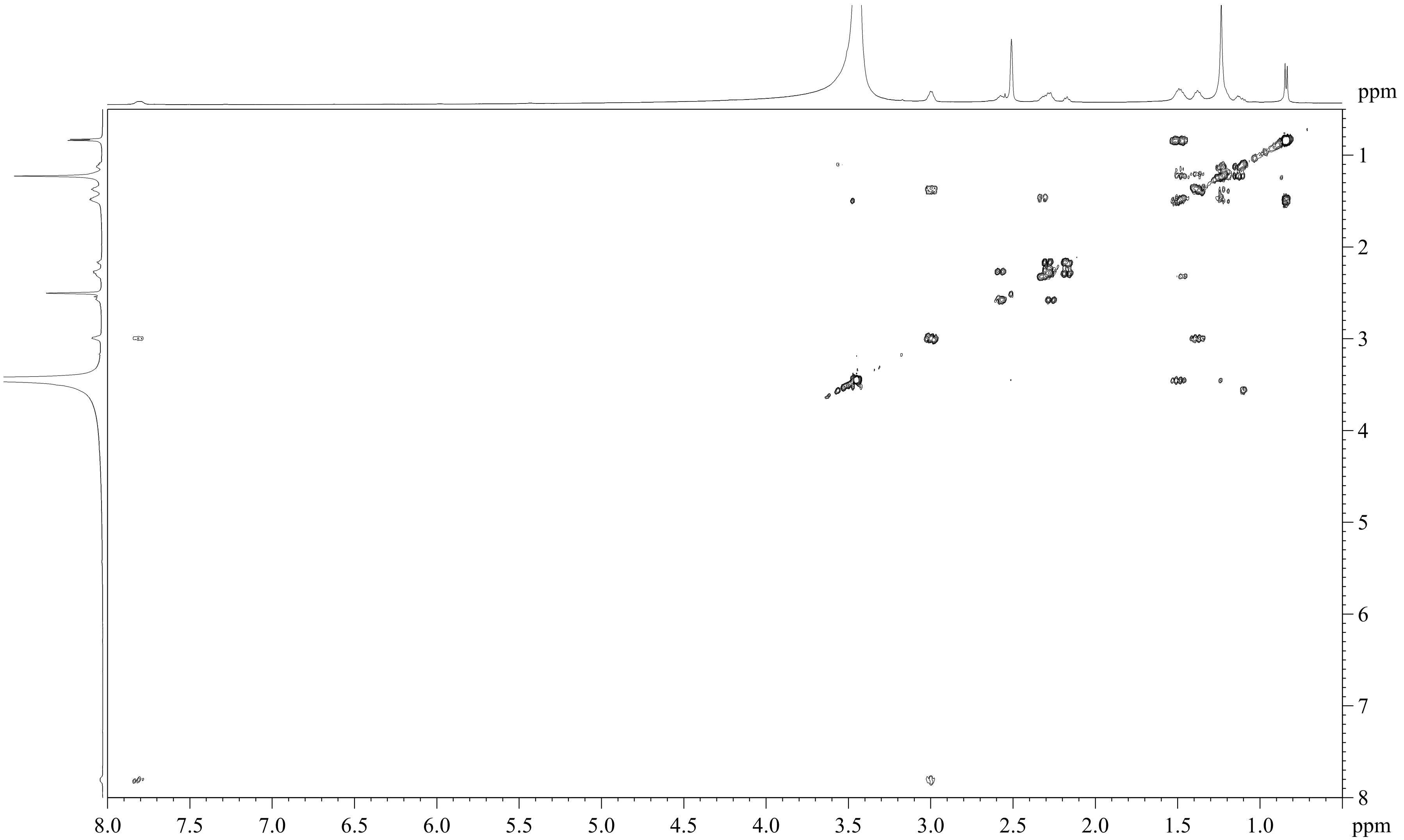

Figure S25: COSY spectrum of **3** (500 MHz, DMSO-d_6, 25 °C).

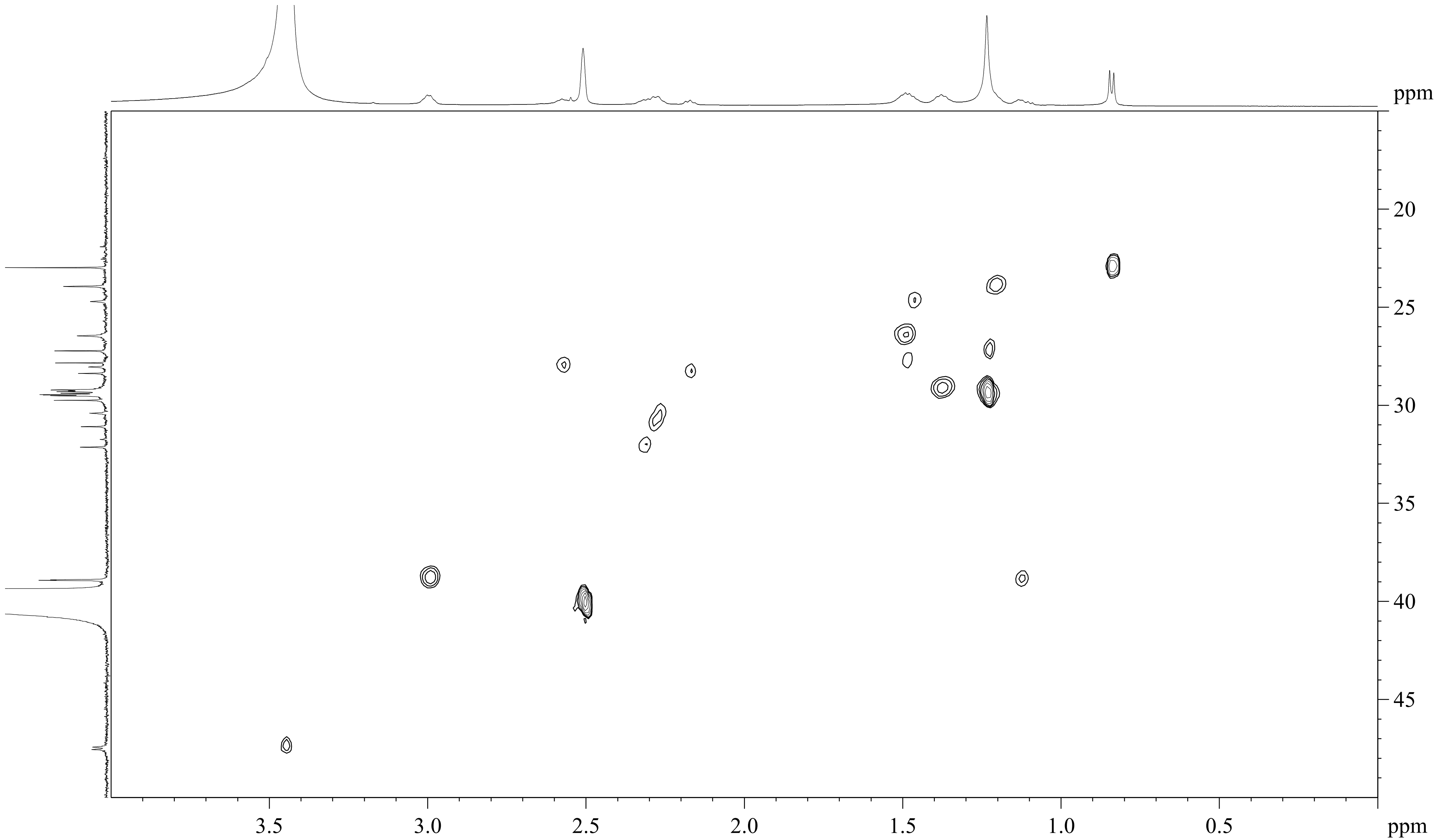

Figure S26: HSQC spectrum of **3** (500 MHz, DMSO-d_6, 25 °C).

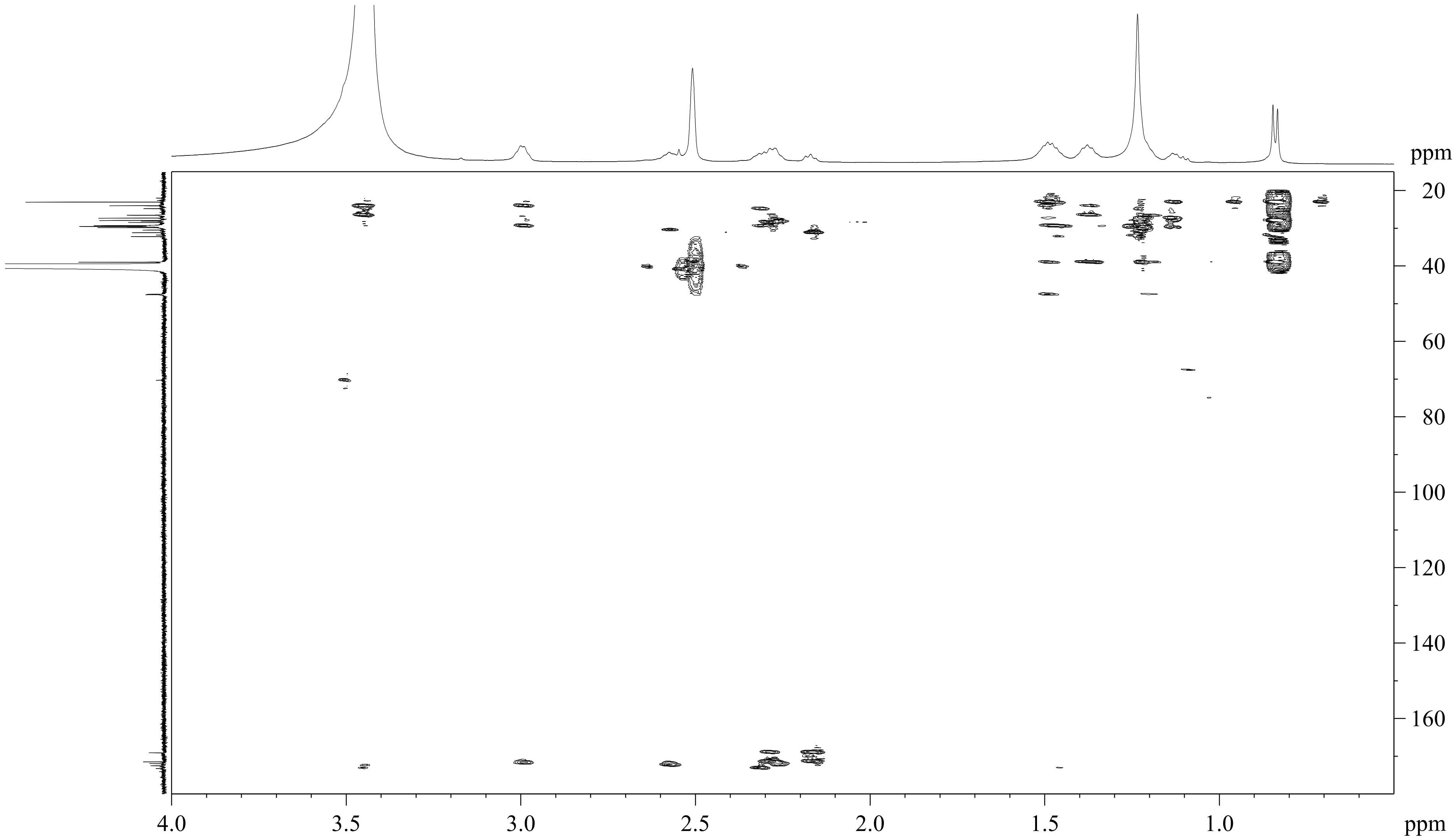

Figure S27: HMBC spectrum of **3** (500 MHz, DMSO-d_6, 25 °C).

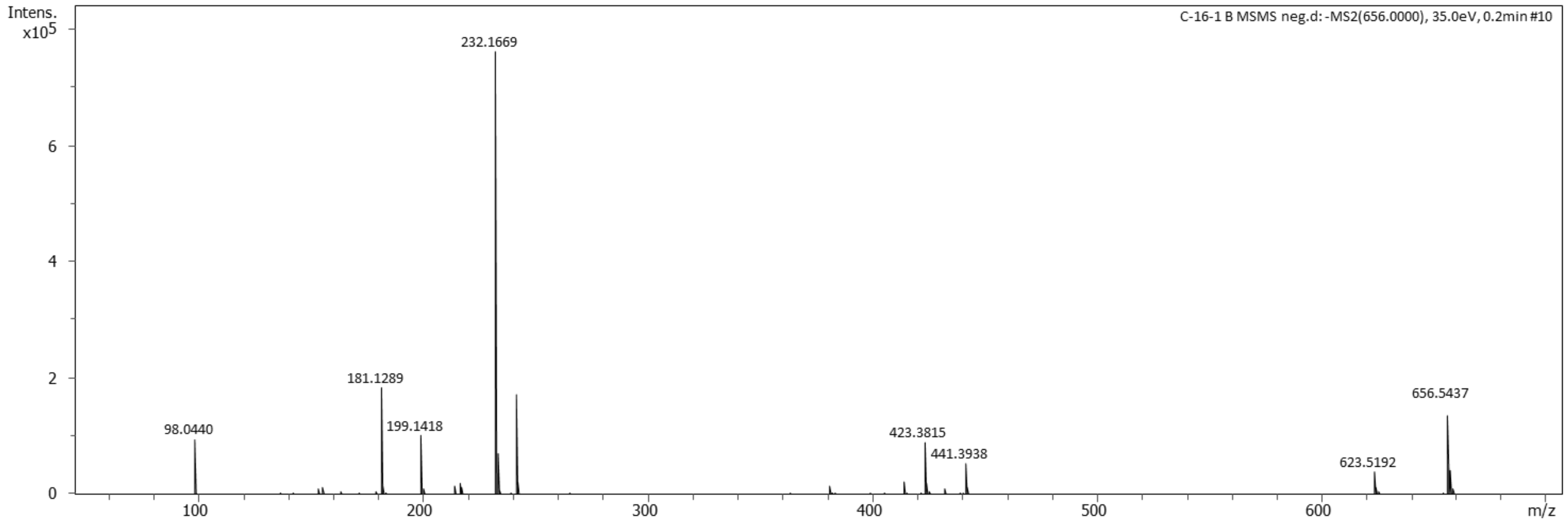

Figure S28: MS/MS spectrum of **3**.

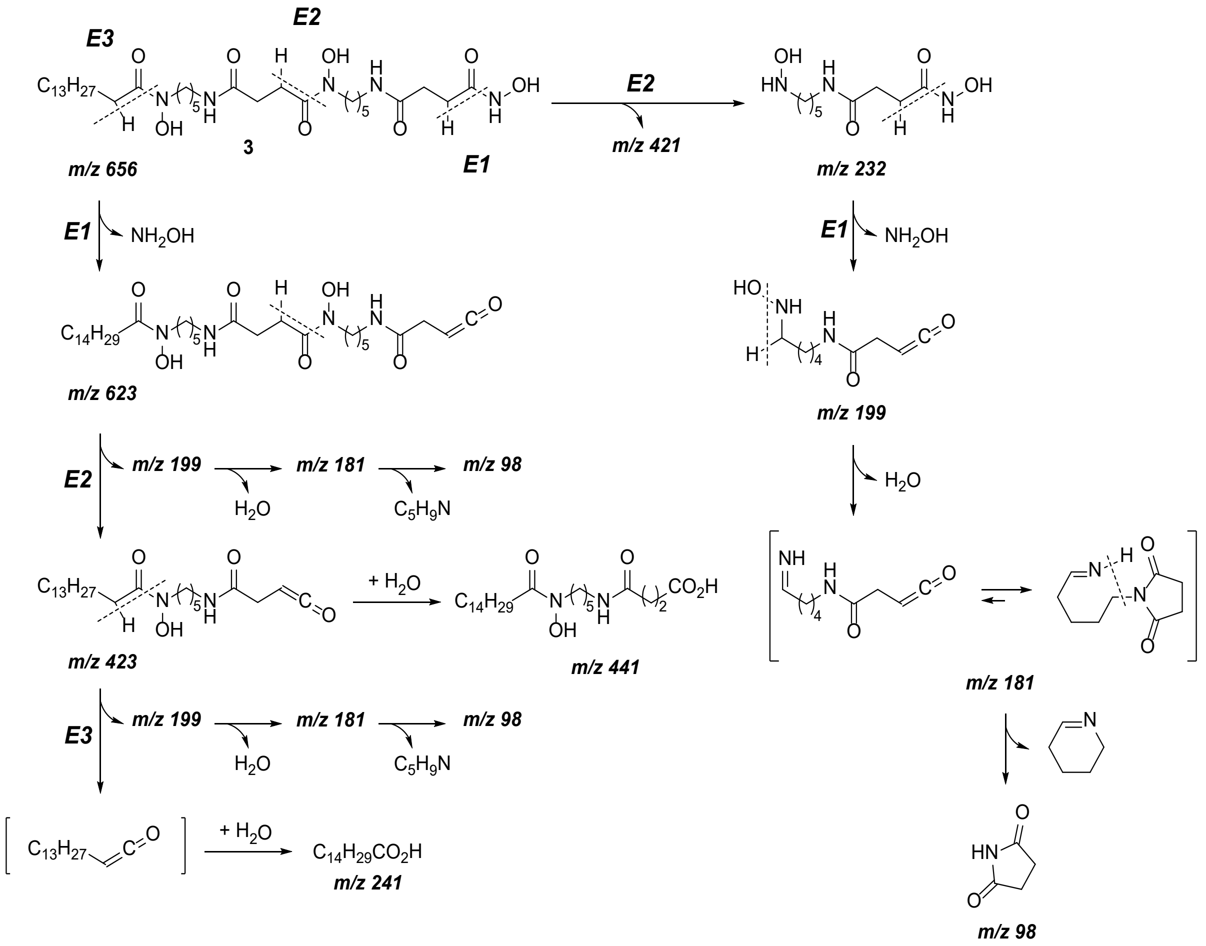

Scheme S29: Fragmentation pathway for **3**.
